# Cell surface SARS-CoV-2 nucleocapsid protein modulates innate and adaptive immunity

**DOI:** 10.1126/sciadv.abp9770

**Published:** 2022-08-03

**Authors:** Alberto Domingo López-Muñoz, Ivan Kosik, Jaroslav Holly, Jonathan W. Yewdell

**Affiliations:** Cellular Biology Section, Laboratory of Viral Diseases, NIAID (NIH), Bethesda, MD, USA.

## Abstract

SARS-CoV-2 nucleocapsid protein (N) induces strong antibody (Ab) and T cell responses. Although considered to be localized in the cytosol, we readily detect N on the surface of live cells. N released by SARS-CoV-2–infected cells or N-expressing transfected cells binds to neighboring cells by electrostatic high-affinity binding to heparan sulfate and heparin, but not other sulfated glycosaminoglycans. N binds with high affinity to 11 human chemokines, including CXCL12β, whose chemotaxis of leukocytes is inhibited by N from SARS-CoV-2, SARS-CoV-1, and MERS-CoV. Anti-N Abs bound to the surface of N-expressing cells activate Fc receptor–expressing cells. Our findings indicate that cell surface N manipulates innate immunity by sequestering chemokines and can be targeted by Fc-expressing innate immune cells. This, in combination with its conserved antigenicity among human CoVs, advances its candidacy for vaccines that induce cross-reactive B and T cell immunity to SARS-CoV-2 variants and other human CoVs, including novel zoonotic strains.

## Teaser

SARS-CoV-2 nucleocapsid protein is secreted, transferred to neighboring cells, inhibits chemotaxis, and enables Ab-based immunity.

## INTRODUCTION

Despite the unprecedented expeditious development and deployment of highly effective vaccines, the rapid selection of severe acute respiratory syndrome coronavirus 2 (SARS-CoV-2) spike glycoprotein (S) antibody (Ab) escape mutants threatens to delay the return to pre-pandemic conditions. To broaden vaccination and reduce SARS-CoV-2–related acute and chronic disease, it is crucial to improve our knowledge of innate and adaptive immunity to CoVs.

CoVs encode four major structural proteins. S, membrane (M), and envelope (E) proteins are localized in the viral surface envelope. N binds to viral RNA through electrostatic interactions, forming cytoplasmic helical nucleocapsids that associate with M to enable virus budding into early secretory compartments. As the most abundantly expressed SARS-CoV-2 protein, N induces strong Ab and T_CD8+_ immune responses (*1*, *2*). Although CoV N is widely considered to be strictly localized in the cytoplasm, cell surface expression of RNA viruses N is more the rule than the exception. Early studies with monoclonal Abs (mAbs) reported surface expression of influenza A and vesicular stomatitis virus N (*3*, *4*). Influenza N is a target for Ab complement–mediated cell lysis (*3*) and Ab-redirected T cell lysis (*5*) and is targeted by protective Abs in mice (*6*). N and N-like RNA genome binding proteins are expressed on the surface of cells infected with other human viruses, including measles (*7*), respiratory syncytial (*8*), lymphocytic choriomeningitis (*9*), and human immunodeficiency virus (*10*). Here, we examine the expression of human CoV N on the cell surface and its participation in innate and adaptive immunity.

## RESULTS

### SARS-CoV-2 N is robustly expressed on the infected cell surface

**Fig. 1.**
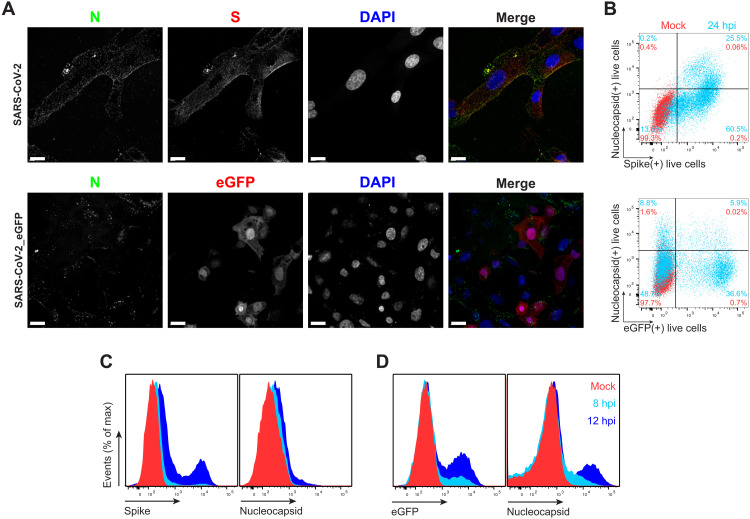
SARS-CoV-2 N is expressed on the surface of live cells early during infection. (**A**) Maximum intensity projections of laser confocal microscopy *z*-stack images of infected Vero cells with wt SARS-CoV-2 (top) or SARS-CoV-2_eGFP, stained live at 24 hpi (MOI = 1). Scale bars, 20 μm. Images are representative of at least three independent experiments with similar results. DAPI, 4′,6-diamidino-2-phenylindole. (**B**) Flow cytometry analyses of Vero cells inoculated with wt (top) or eGFP-expressing (bottom) SARS-CoV-2 (MOI = 1), stained live at 24 hpi against SARS-CoV-2 S and N proteins. Representative dot plots of flow cytometry analyses showing double staining of surface S, N, and eGFP proteins, indicating the percentage of the gated cell population for each quadrant of the double staining. Data are representative of at least three independent experiments, each performed with triplicate samples. (**C** and **D**) Time course of surface S, N, and eGFP protein expression in live infected Vero cells with wt (C) and eGFP reporter (D) SARS-CoV-2 at 8 and 12 hpi (MOI = 1). Representative histogram overlays of surface S, N, and intracellular eGFP proteins of flow cytometry analyses. Data are representative of one experiment of at least two independent experiments performed in triplicate.

We examined cell surface expression of SARS-CoV-2 N by imaging Vero cells 24 hours post-infection (hpi) with wild-type (wt) or a recombinant SARS-CoV-2 expressing enhanced green fluorescent protein (SARS-CoV-2_eGFP). To exclusively detect cell surface N, we incubated live cells with primary and fluorophore-conjugated secondary Abs at 4°C before fixation and mounting for confocal imaging. This revealed clear surface N staining over mock-infected (mock) background levels, using S or eGFP as markers of infected cells (Fig. 1A, maximum intensity projection images of *z* stack). We similarly found N on the surface of BHK-21_hACE2 (human angiotensin-converting enzyme 2), Caco-2, Calu-3, CHO-K1_hACE2, and HEK293-FT_hACE2 cells infected with wt or eGFP SARS-CoV-2 at 24 hpi (figs. S1 and S2, movies S1 to S14, and animations S1 to S14). Depending on the cell type, we observed a variable degree of colocalization between N and S, particularly remarkable in Vero (Fig. 1A), Calu-3, CHO-K1_hACE2, and HEK293-FT_hACE2 cells (fig. S1). We noted a marked syncytia formation in hACE2-overexpressing BHK-21_hACE2 and HEK293-FT_hACE2 cells as reported (*11*).

To measure N surface expression more quantitatively, we performed flow cytometry analyses of live infected cells 24 hpi. Surface N was detected on a subpopulation of S- or eGFP-expressing cells for each of the seven cell types examined (Fig. 1B and figs. S1 to S3). N was also detected on the surface of live cells infected with Alpha (B.1.1.7), Beta (B.1.351), and Delta (B.1.617.2) SARS-CoV-2 variants (fig. S4). Via flow cytometry, we determined the kinetics of N expression on the surface of Vero (Fig. 1, C and D), BHK-21_hACE2, and A549_hACE2 cells (fig. S5). As early as 8 hpi, we observed a significant surface signal for N protein in live Vero and BHK-21_hACE2–infected cells, while it took slightly longer (12 hpi) for A549_hACE2 cells (fig. S5). Notably, depending on cell type and marker of infection (S versus eGFP), we detected cells expressing N but not S or eGFP on a fraction of cells, ranging from less than 1 to 43% (figs. S1 and S2). This is consistent with several mechanisms acting alone or in combination: differential expression of SARS-CoV-2 gene products in individual cells (*12*), complete retention of S in the secretory pathway (*13*), and transfer of N from infected to noninfected cells.

**Fig. 2.**
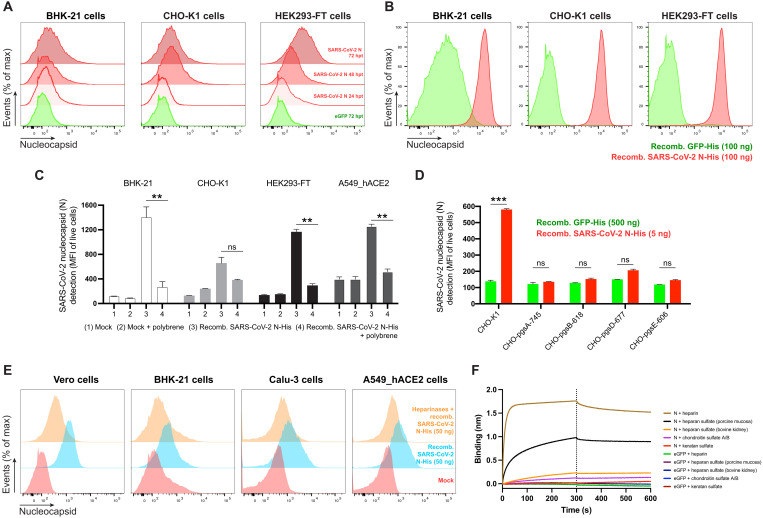
SARS-CoV-2 N cell surface binding is independent of other viral genes and is specifically mediated by heparan sulfate/heparin. (**A**) Histogram semi-overlays of kinetics of surface N protein expression in cells transiently transfected with a plasmid encoding eGFP or N protein, detected with Abs by flow cytometry. hpt, hours post-transfection. (**B**) Histogram overlays of analyses of exogenous rN binding to different cells, incubated with purified eGFP or rN protein for 15 min, stained with Abs, and analyzed by flow cytometry. (**C**) Electric charge neutralization assays with a cationic polymer (polybrene). Cells were incubated with 50 ng of rN protein for 15 min and then with polybrene (10 μg/ml), stained with Abs, and analyzed. (**D**) Different GAG-deficient CHO cells were incubated with eGFP or rN protein for 15 min, stained with Abs, and analyzed by flow cytometry. (**E**) Heparinase treatment significantly abrogates the cell ability to bind and retain N protein. Histogram semi-overlays of different cells treated with heparinases for 1 hour, incubated with 50 ng of rN protein for 15 min, stained with Abs, and analyzed. (**F**) BLI sensorgrams from high-throughput screening binding assays of sulfated GAGs to immobilized N or eGFP proteins. SA-coated biosensors were loaded with equivalent amounts of N or eGFP, measuring their ability to bind each GAG. Sensorgrams show association and dissociation phases, where the vertical dotted line indicates the end of the association step. In (C) and (D), the mean fluorescent intensity (MFI) of detected surface N protein from live cells is plotted, showing means ± SEM (*n* = 2). Student’s two-tailed unpaired *t* test was used to compare N-incubated cells versus N-incubated and polybrene-treated cells (C) and to compare GFP- versus N-incubated cells (D): ns (statistically nonsignificant) *P* > 0.01, **P < 0.01, and ***P < 0.001. The analyses were repeated with different protein preparations, and one representative assay of at least three independent assays performed in duplicate is shown.

To determine whether N cell surface expression requires other SARS-CoV-2 gene products, we transfected cells with an expression plasmid encoding N. Staining of live BHK-21, CHO-K1, or HEK293-FT cell transfectants revealed up to sevenfold more N mAb binding over background levels obtained with cells transfected with a control plasmid expressing eGFP (Fig. 2A and fig. S6A). N surface expression increased between 24 and 72 hours after transfection, providing further evidence for the specificity of staining and demonstrating that cell surface expression is an intrinsic property of biosynthesized N.

### Exogenous N binds to cells

To examine whether N surface expression requires its synthesis in the cell, we incubated BHK-21, CHO-K1, or HEK293-FT cells with exogenous purified recombinant N (rN) for 15 min at 37°C. This resulted in strong flow cytometry staining (2 log shift) with anti-N mAbs relative to control cells incubated with an irrelevant protein (Fig. 2B and fig. S6B).

N interacts with negatively charged viral RNA through highly positively charged RNA binding domains (*14*, *15*). We examined charge-based N binding to the cell surface by treating rN-coated cells with polybrene, a cationic polymer that neutralizes surface electrostatic charges. Flow cytometry analysis showed that polybrene decreases rN bound to live BHK-21, CHO-K1, HEK293-FT, and A549_hACE2 cells to similar levels, with the magnitude of the effect proportional to the amount of bound N (Fig. 2C).

For most mammalian cells, glycosaminoglycans (GAGs) are the major negatively charged molecule on the plasma membrane (*16*). To assess the contribution of GAGs to N cell surface binding, we used a panel of GAG-deficient CHO cells (*17*). Each of the GAG-deficient cell lines tested failed to bind rN over levels observed with recombinant GFP (Fig. 2D). The panel included cells with the complete absence of GAGs (CHO-pgsA-745 and CHO-pgsB-618) and cells with defects in synthesizing heparan sulfate and heparin but no other GAGs (CHO-pgsD-677 and CHO-pgsE-606). Consistent with these findings, treating Vero, BHK-21, Calu-3, A549_hACE2, CHO-K1, or HEK293-FT cells with heparinase I, II, and III in combination, to depolymerize heparan sulfate/heparin polysaccharide chains to disaccharides, cells markedly reduced binding of exogenous rN (Fig. 2E and fig. S6C). We directly confirmed N binding to heparin by using biolayer interferometry (BLI), where we directly demonstrate N-specific nanomolar affinity binding to heparan sulfate and heparin but not to other sulfated GAGs (Fig. 2F and fig. S6, D and E). Together, these findings indicate that N binds to the cell surface by interacting with heparan sulfate and heparin in a charge-dependent manner.

### N is transferred from expressing to nonexpressing cells

**Fig. 3.**
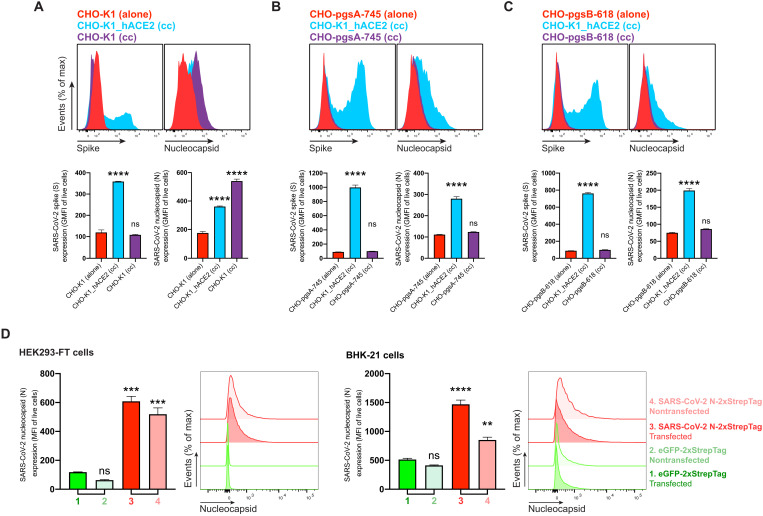
SARS-CoV-2 N protein is transferred to the cell surface of neighboring uninfected cells. Flow cytometry analyses of N transfer assays between donor and recipient cocultured cells. (**A** to **C**) N transfer assays between infectable and noninfectable cocultured cells. CHO-K1 (A), GAG-deficient CHO-pgsA-745 (B), and CHO-pgsB-618 (C) cells (noninfectable), alone or cocultured (cc) with CHO-K1_hACE2 cells (infectable), were inoculated with SARS-CoV-2 (MOI = 1) and stained live with Abs at 24 hpi against surface SARS-CoV-2 S and N proteins. Noninfectable cells were stained with CellTrace Violet before coculture with infectable cells (fig. S8). For dot plots showing double staining of surface S and N with percentages of the gated cell population for each quadrant see fig. S8 (C to E). (**D**) N transfer assays between transfected and nontransfected cocultured cells. HEK293-FT and BHK-21 cells were transiently transfected with a plasmid encoding eGFP or N protein. After 24 hours, nontransfected HEK293-FT or BHK-21 cells were stained with CellTrace Violet before being added and cocultured with their transfected counterparts. Cells were stained live after 24 hours with Abs and analyzed. For each assay, the following is shown: Histogram overlays and semi-overlays of surface N and S proteins, as well as the geometric MFI (GMFI) or MFI of live cells expressing S and N proteins, are plotted, showing means ± SEM (*n* = 3). One representative experiment of at least three independent experiments performed in triplicate is shown. One-way analysis of variance (ANOVA) and Dunnett’s multiple comparisons test were used to compare all conditions against noninfectable cells cultured alone within each assay or against eGFP-transfected cells: ns *P* > 0.05, **P < 0.01, ***P < 0.001, and ****P < 0.0001.

In SARS-CoV-2 immunofluorescence and flow cytometry experiments at 24 hpi (Fig. 1, A and B, and figs. S1 and S2), we observed cells expressing N but not S or GFP as early as 8 and 12 hpi (fig. S5), with increasing numbers of cells over time after infection (fig. S7). To determine whether N can be transferred from infected to noninfected cells, we added SARS-CoV-2 to a coculture of infectable (hACE2-expressing) and noninfectable (non–hACE2-expressing) CHO-K1 cells, at a ratio of 1 infectable to 9 noninfectable cells. We confirmed that hACE2 is required for infecting CHO cells with SARS-CoV-2 (fig. S8A). We prestained noninfectable cells with CellTrace Violet to enable unambiguous flow identification after coculture (fig. S8B). Cocultured non-ACE2–expressing uninfected CHO-K1 cells (also confirmed by the absence of S expression) had a higher cell surface N signal than infected cells (Fig. 3A and fig. S8C). N transfer from infected cells required GAG expression on uninfected cells, as shown by near background staining by GAG-deficient CHO cells (Fig. 3, B and C, and fig. S8, D and E). N was also transferred from HEK293-FT or BHK-21 cells transiently expressing N from a transgene to cocultured untransfected cells (Fig. 3D). Extending these findings, we found that N from SARS-CoV-2–infected cells is transferred to uninfectable cells separated by a 3-μm pore size filter, conclusively establishing that cell-cell contract is not required for transfer to the cell surface (fig. S9). On the basis of these findings, we conclude that N protein synthesis leads to its release from cells and its robust transfer, at least partially by diffusion, to nonsynthesizing cells, where it is retained on the cell surface by binding heparin/heparan sulfate.

### SARS-CoV-2 N inhibits chemokine function but enables Ab-based immune cell activation

**Fig. 4.**
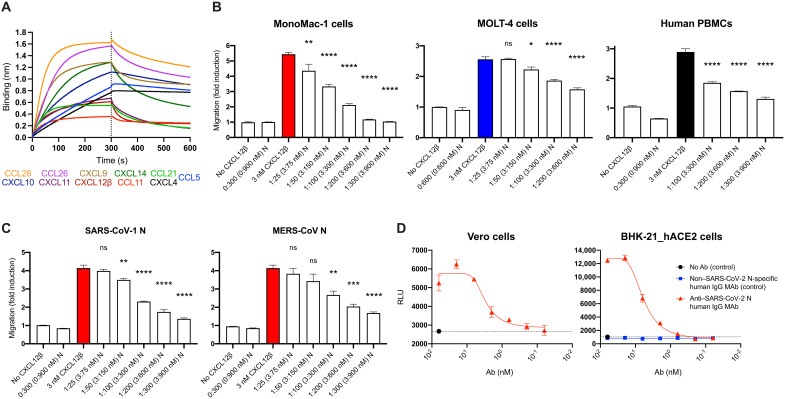
SARS-CoV-2 N protein modulates innate and adaptive immunity. (**A** and **B**) N binds chemokines through their GAG-binding domain and inhibits in vitro chemokine-mediated leukocyte migration. (A) BLI sensorgrams of binding assays showing association and dissociation phases of the interaction between N protein and 11 positively bound chemokines at a concentration of 100 nM of 64 human cytokines tested. The dotted line indicates the end of the association step. The analyses were repeated with different purified rN protein preparations. One representative assay of three independent assays is shown. (B) SARS-CoV-2 N blocks CXCL12β chemotaxis of MonoMac-1 cells, MOLT-4 cells, and human PBMCs. (**C**) SARS-CoV-1 N and MERS-CoV N block CXCL12β chemotaxis of MonoMac-1 cells. CXCL12β was incubated alone or in the presence of the indicated viral protein in the lower chamber of transwell migration devices. Migrated cells from the top chamber were detected in the lower chamber at the end of the experiment. The induction of migration shows means ± SEM (*n* = 3) from one representative assay performed in triplicate of at least three independent assays. One-way ANOVA and Dunnett’s multiple comparisons test were used to compare all conditions (except controls) against the migration induced by the chemokine alone (colored bars): ns *P* > 0.05, *P < 0.05, **P < 0.01, ***P < 0.001, and ****P < 0.0001. (**D**) N protein is a target for Ab-based immunity. ADCC reporter bioassays were performed on SARS-CoV-2–infected Vero and BHK-21_hACE2 target cells (24 hpi, MOI =1) using decreasing concentrations of a human mAb against the N protein and Jurkat effector reporter cells. After overnight incubation, luciferase expression to gauge cell activation was measured. Data show means ± SEM (*n* = 3) of one representative assay of three independent experiments performed in triplicate. Dashed lines show background signal detected in the absence of Ab. RLU, relative luciferase units.

The robust expression of N on the surface of infected and surrounding cells suggests a significant evolutionary function. SARS-CoV-2, like most viruses, induces the release of proinflammatory cytokines by infected cells. Could N interfere with this signaling? We examined the ability of immobilized N to interact with 64 human cytokines by BLI. N bound CCL5, CCL11, CCL21, CCL26, CCL28, CXCL4, CXCL9, CXCL10, CXCL11, CXCL12β, and CXCL14 chemokines with micromolar to nanomolar affinities (Fig. 4A and fig. S10). By contrast, none of the other SARS-CoV-2–immobilized structural (S, M, and E) or nonstructural [open reading frames (ORFs) 3a, 3b, 6, 7a, 7b, 8, 9b, 9c, and 10] proteins tested interacted with any of the cytokines in the panel with affinities higher than that observed for immobilized GFP (fig. S11A). Kinetic curves of N binding to each chemokine were biphasic, deviating from first-order binding (1:1) and showing binding heterogeneity (fig. S10) (*18*).

Chemokine function is based on interaction with both surface GAGs and their specific receptors located in the surface of leukocytes. We found that blocking the GAG-binding domain of chemokines by increasing concentrations of heparan sulfate (from bovine kidney) and chondroitin sulfate A and B N binding to its subset of chemokines was abrogated (fig. S12). This indicates that N binds to chemokines through their GAG-binding domain.

What is the functional relevance of N-chemokine interactions? Transwell chemotaxis experiments with monocyte-like cells (MonoMac-1), T lymphocyte–like cells (MOLT-4), and human healthy donor peripheral blood mononuclear cells (PBMCs) revealed that rN blocked CXCL12β-induced chemotaxis at physiologically relevant concentrations (Fig. 4B). We estimated the amount of physiologically released N during infection by comparing surface fluorescence between infected cells 24 hpi (fig. S3) and cells incubated with 1 μM (50 ng) of rN (Fig. 2E and fig. S6C), observing a similar gain in surface fluorescent signal by anti-N Ab staining performed in identical conditions. Thus, the near-complete rN inhibition of chemokine-induced migration at 300 nM occurs at a threefold lower concentration than that needed to match the amount of cell surface N routinely observed following SARS-CoV-2 infection. While rN from different vendors showed comparable results inhibiting CXCL12β-mediated induction of migration of MonoMac-1 cells, the S1 protein (subunit 1 of the S protein, containing the receptor-binding domain) had no inhibitory effect on CXCL12β-induced chemotaxis (fig. S13). Extending these findings, rN from both SARS-CoV-1 and Middle East respiratory syndrome (MERS)–CoV also inhibited CXCL12β-induced migration of MonoMac-1 cells (Fig. 4C).

mAbs to mouse CoV N have been reported to exert antiviral activity in vitro (with complement) and in vivo (*19*, *20*). To examine their potential as a target for Ab-dependent cellular cytotoxicity (ADCC), we used FcγRIIIa receptor–expressing Jurkat reporter cells as a surrogate for ADCC effector cell recognition of anti-N mAb–coated SARS-CoV-2–infected cells. Vero and BHK-21_hACE2 SARS-CoV-2–infected cells activated reporter cells in an anti-N mAb concentration–dependent manner (Fig. 4D). Activation was not observed in the absence of infection or with a control human mAb with the same heavy chain.

Together, these findings indicate that N protein from each of the highly pathogenic human CoVs blocks chemokine function. This is consistent with the possibility that cell surface N blocks chemokine function in vivo, facilitating viral replication and transmission. Conversely, we also show that cell surface N is a potential target for ADCC, which potentially contributes to limiting viral replication and transmission.

## DISCUSSION

Here, we show that N synthesized during SARS-CoV-2 infection or from a transfected complementary DNA is expressed on the surface of both N synthesizing cells and neighboring cells. N binding to the cell surface is based on specific association with heparan sulfate and heparin. The most parsimonious explanation for these findings is that N is released from cells and binds to both producing and bystander cells from liquid phase. On the basis of the flow cytometry data, levels of N on SARS-CoV-2–infected cells equals or exceeds cell surface S on all but one of the seven cell types examined. This, in part, is due to the retention of a substantial fraction of S in the early secretory pathway but also reflects a robust amount of cell surface N, likely in the range of 10^4^ to 10^5^ copies per cell.

The mechanism underlying N secretion remains to be established. N has two potential sites for addition of N-linked oligosaccharides in the secretory pathway that are glycosylated and readily detected when N is targeted to the endoplasmic reticulum (ER) with an artificial N-terminal signal sequence (*21*). Without a signal sequence, N is not glycosylated. This indicates that, as with other viral nucleic acid–binding proteins (e.g., SV40 T antigen and influenza virus N), SARS-CoV-2 N is likely secreted through a noncanonical secretory pathway, possibly one of the three defined pathways that bypass insertion into the ER (*22*). Like N, several proteins noncanonically exported to the cell surface (HIV Tat, FGF2, and tau) bind heparan sulfate, which has been shown to be involved in traversing the plasma membrane (*23*). It will be interesting to examine the extent to which cell surface export of N and other viral RNA binding proteins, as well as their cell surface binding, is based on heparan sulfate association.

N is typically the most abundantly expressed SARS-CoV-2 protein, and its transfer to noninfected cells potentially amplifies its contributions to viral fitness. The remarkable ability of cell surface N to bind chemokines and block chemotaxis of immune effector cells offers an evolutionary explanation for its cellular export and binding to infected and neighboring uninfected cells. Like N, chemokines are immobilized on source cells and their neighbors by binding to GAGs. A number of viruses are known to express chemokine-binding proteins, which modulate chemokine activity by interacting with the GAG- or receptor-binding domain of chemokines or both (*24*, *25*). Our findings establish SARS-CoV-2 N as the first CoV chemokine binding protein, one with a remarkably high affinity (nanomolar range) for multiple chemokines.

The binding of N to heparin, which limits coagulation at inflammation sites, suggests a possible role for secreted N in promoting coronavirus disease (COVID)–associated clotting abnormalities. N is present in intestine and lungs from recovered and fatal patients with COVID-19, respectively, while virus-like particles are rarely detected (*26*, *27*), consistent with this intriguing possibility and a role in the chronic low-level inflammation that causes “long COVID-19” symptoms.

The remarkable efficacy of spike-only vaccines demonstrates that Abs to N are not required for COVID-19 protection. SARS-CoV-2 induces a robust anti-N Ab response, in part, likely due to cross-reaction with memory B cells induced by seasonal CoV infections. These Abs may reduce SARS-CoV-2 disease in naïve individuals since we establish N as a potential target for Ab-mediated effector functions, including complement and natural killer cell–mediated lysis of infected cells. Abs, therefore, may play an unexpected role in protection to SARS-CoV-2 infection afforded by immunization with N-expressing vectors presumed to function via induction of N-specific T cells (*28*–*31*). N is an attractive vaccine target due to its strong immunogenicity and much lower antigenic drift than spike. This may be particularly relevant given the remarkable capacity of SARS-CoV-2 to acquire amino changes in S as illustrated by the recent introduction of the omicron variant with more than 30 nonsynonymous mutations in S.

In summary, our findings demonstrate unexpected roles for N in innate and adaptive immunity to SARS-CoV-2 and other human CoVs that may contribute to both pathogenesis and protection and support N as an Ab and T cell target for future “universal” vaccines that provide broad protection against both future strains of SARS-CoV-2 and other human CoVs.

## MATERIALS AND METHODS

### Cells

Vero cells (no. CCL-81), BHK-21 (no. CCL-10), Caco-2 (no. HTB-37), Calu-3 (no. HTB-55), CHO-K1 (no. CCL-61), CHO-pgsA-745 (no. CRL-2242), CHO-pgsB-618 (no. CRL-2241), CHO-pgsD-677 (no. CRL-2244), CHO-pgsE-606 (no. CRL-2246), HEK293-FT (no. CRL-11268), A549 (no. CCL-185), and MOLT-4 (no. CRL-1582) cells were from the American Type Culture Collection. MonoMac-1 cells (no. ACC 252) were from the German Collection of Microorganisms and Cell Cultures. PBMCs were obtained from a healthy donor with informed consent, at the Department of Transfusion Medicine [National Institutes of Health (NIH)]. Vero, BHK-21, Caco-2, Calu-3, and HEK293-FT cells were grown in Dulbecco’s modified Eagle’s medium (DMEM) with GlutaMAX (Thermo Fisher Scientific, no. 10566016). CHO-K1, CHO-pgsA-745, CHO-pgsB-618, CHO-pgsD-677, CHO-pgsE-606, and A549 cells were grown in F-12K medium (Thermo Fisher Scientific, no. 21127022). PBMCs, MOLT-4, and MonoMac-1 cells were grown in RPMI 1640 (Thermo Fisher Scientific, no. 11875119). BHK-21_hACE2, CHO-K1_hACE2, HEK293-FT_hACE2, and A549_hACE2 cells were grown in their correspondent medium with blasticidin (250 to 500 μg/ml; InvivoGen, no. ant-bl-1). All cell media were supplemented with 8% (v/v) not heat-inactivated fetal bovine serum (FBS) (HyClone, no. SH30071.03) but Caco-2 with 20%, and cells were grown cultured at 37°C with 5% CO_2_ in sterile flasks. Cells were passaged at ~80 to 90% confluence and seeded as explained for each individual assays.

### SARS-CoV-2 stock preparation

SARS-CoV-2 (isolate USA-WA1/2020, no. NR-52281), SARS-CoV-2_eGFP (no. NR-54002), and the Alpha variant (B.1.1.7, no. NR-54000) were obtained from BEI Resources. The Beta (B.1.351) and Delta (B.1.617.2) variants were obtained from A. Pekosz (Johns Hopkins University, USA). Viruses were propagated by the National Institute of Allergy and Infectious Diseases (NIAID) SARS-CoV-2 Virology Core Laboratory under biosafety level 3 (BSL-3) conditions using Vero (CCL-81) or Vero-overexpressing human TMPRSS2 cells, cultured in DMEM supplemented with GlutaMAX, 2% FBS, penicillin, streptomycin, and fungizone. Virus stocks were deep-sequenced and subjected to minor variants analysis by the NIAID SARS-CoV-2 Virology Core Laboratory. The median tissure culture infectious dose and plaque-forming units (PFU) of virus in clarified culture medium were determined on Vero cells after staining with crystal violet. SARS-CoV-2 infections were performed in the NIAID SARS-CoV-2 Virology Core BSL-3 laboratory strictly adhering to its standard operative procedures.

### Generation of mutant cell lines constitutively expressing hACE2

The Sleeping Beauty transposase system was used for the generation of BHK-21_hACE2, CHO-K1_hACE2, HEK293-FT_hACE2, and A549_hACE2 cells as previously described (*32*, *33*). Briefly, a semi-confluent 60-mm plate was seeded with each cell line and cotransfected with 0.5 μg of pCMV(CAT)T7-SB100 (transposase vector; Addgene, no. 34879) and 5 μg of pSBbi-Bla hACE2 (transposon vector), using TransIT-LT1 Transfection Reagent (Mirus Bio), following the manufacturer’s instructions. After 24 hours, cells were transferred to a T-75 flask and selected with blasticidin (250 to 500 μg/ml) for 2 weeks. The surface expression of hACE2 was confirmed by flow cytometry using anti-human ACE2 Alexa Fluor 647–conjugated Ab (R&D Systems, no. FAB9332R). The expression was further confirmed by immunoblot using hACE2 Ab (Cell Signaling Technology, no. 4355). The ORF of hACE2 (provided by S. Best from NIAID/NIH) was cloned into pSBbi-Bla vector (Addgene, no. 60526) as described (*32*).

### Antibodies

Previously published Ab VH and VL amino acid sequences against SARS-CoV-2 N [#N18 in (*34*)] and SARS-CoV-2 S [#H4 in (*35*)] were commercially synthesized, cloned into a human immunoglobulin G1 (IgG1) vector backbone, produced, and purified (Synbio Technologies). A 100-μg aliquot of anti–SARS-CoV-2 N human mAb was conjugated with the Alexa Fluor 647 Lightning-Link Conjugation Kit (Abcam, no. ab269823), while 100 μg of anti–SARS-CoV-2 S was conjugated with the Alexa Fluor 488 Lightning-Link Conjugation Kit (Abcam, no. ab236553). Each experiment was repeated with similar results using anti–SARS-CoV-2 N rabbit polyclonal Ab (GeneTex, no. GTX135357) and anti–SARS-CoV-2 S rabbit polyclonal Ab (ProSci, no. 3525). Goat anti-mouse IgG Alexa Fluor 488 (Thermo Fisher Scientific, no. A-11001) or 647 (no. A-21235), goat anti-rabbit IgG Alexa Fluor 488 (no. A-11008) or 647 (no. A-21245), and goat anti-human IgG Alexa Fluor 488 (no. A-11013) or 647 (no. A-21445) were used as a secondary Abs.

The SARS-CoV-2 S stabilized (S^st^) sequence (*36*) (R710G, R711S, R713S, K1014P, and V1015P) was commercially cloned into a mammalian expression vector, produced, and purified (GenScript Biotech). Mouse polyclonal anti–SARS-CoV-2 S^st^ serum was produced as follows: 8- to-12-week C57B6 mice (Taconic Farms Inc.) were immunized with 4 μg of S^st^ diluted in Dulbecco’s phosphate-buffered saline (DPBS), adjuvanted by TiterMax Gold (MilliporeSigma, no. T2684) (2:1) in 50-μl volume via intramuscular injections. Serum was collected 21 days after booster immunization, heat-inactivated at 56°C for 30 min, aliquoted, and stored at 4°C. Abs and serum were titrated, and specificity was tested, by flow cytometry on HEK293-FT cells transiently expressing SARS-CoV-2 N (Addgene, no. 141391), S (BEI Resources, no. NR-52310), or S^st^.

### Immunofluorescence

For confocal microscopy imaging, 2.5 × 10^4^ cells were seeded on 12-mm glass coverslips in 24-well plates in indicated medium with gentamycin (25 μg/ml) overnight. Cells were infected with SARS-CoV-2 at a multiplicity of infection (MOI) of 1 PFU per cell for 1 hour at 37°C. Virus was aspirated, and cells were then incubated in cell growth medium. After 24 hours, the cells were washed with DPBS (Gibco, no. 14190-144) containing 5% goat serum (Jackson ImmunoResearch Labs, 005-000-121). Primary and secondary Abs were incubated with live cells at 4°C for 30 min. Cells were then washed twice with DPBS/5% goat serum and fixed in 4% paraformaldehyde (PFA) for 30 min at room temperature. After fixation, coverslips were washed in DPBS and deionized _i_H_2_O and mounted with 4′,6-diamidino-2-phenylindole Fluoromount-G mounting medium (VWR, no. 102092-102). Images were acquired with a Leica STELLARIS 8 confocal microscope platform equipped with ultraviolet and white light lasers, using a 63× oil immersion objective (Leica Microsystems, no. 11513859), with a 1× zoom resolution of 512 × 512 pixels. Maximum intensity projections were processed from *z* stacks (at least 15 *z* steps of 0.3 μm per image), and background correction (Gaussian filter) and color processing were processed using Imaris (Bitplane). Background levels of signal for each cell type were set on the basis of mock-infected stained conditions. Animations (gifs) were generated with Photoshop 2022 (Adobe). Tile scans were taken of representative infected areas, and individual fields (tiles) were merged into one image. Mock-infected coverslips were processed in parallel with infected counterparts, SARS-CoV-2–infected coverslips were also incubated with all secondary Abs only as controls, and images were acquired using identical photomultiplier and laser settings.

### Flow cytometry

For cell surface protein expression analyses, 1 × 10^5^ cells were seeded on 24-well plates and mock or SARS-CoV-2–infected at an MOI of 1 PFU for 1 hour at 37°C, followed by aspirating the virus inoculum and adding medium containing 2% FBS. After the indicated hpi, the cells were washed with DPBS, trypsinized with TrypLE Express Enzyme (Thermo Fisher Scientific, no. 12604039) or 0.25% Trypsin-EDTA (Thermo Fisher Scientific, no. 25200056) for 5 min at 37°C, transferred to a 96-well plate, and washed with Hanks’ balanced salt solution (HBSS) (Lonza, no. 10-527F) with 0.1% bovine serum albumin (BSA). Cells were stained live with Alexa Fluor–conjugated Abs (or primary and secondary Abs) and the LIVE/DEAD Fixable Violet Dead Cell Stain Kit (Thermo Fisher Scientific, no. L34964) in DPBS, for 25 min at 4°C. After Ab staining, cells were washed twice with HBSS containing 0.1% BSA and then fixed in 4% PFA for 30 min at room temperature. PFA was aspirated, and cells were resuspended in HBSS 0.1% BSA for analysis. To control for possible removal of cell surface antigens by trypsinization, in parallel, infected cells after 24 hours were washed with DPBS and directly stained in the 24-well plates before trypsinization. This resulted in similar levels of cell surface viral protein detection between trypsinized-stained and vice versa conditions.

For cell surface protein binding assays using recombinant proteins, indicated cells were trypsinized and washed with DPBS, and 1 × 10^5^ cells were transferred to 96-well plates. Indicated amounts of recombinant GFP-His (Thermo Fisher Scientific, no. A42613) or SARS-CoV-2 N-His (Sino Biological, no. 40588-V08B; Acro Biosystems, no. NUN-C5227; Ray Biotech, no. 230-30164) were resuspended in 100 μl of DPBS, and cells were incubated for 15 min at 37°C and orbital shaking of 150 rpm. Cells were washed twice and stained as described above for subsequent flow cytometry analysis. For electric charge neutralization assays, after incubation with recombinant proteins and being washed twice, cells were incubated with polybrene (10 μg/ml; MilliporeSigma, no. TR-1003-G) in DPBS for 15 min at 37°C and orbital shaking of 150 rpm. Then, cells were washed twice and stained as described above for subsequent analysis.

For heparinase assays, 1 × 10^5^ single cells in 96-well plates were treated with Bacteroides heparinase I (4.8 U), II (1.6 U), and III (0.28 U) (New England Biolabs, nos. P0735S, P0736S, and P0737S) in DPBS for 1 hour at 30°C. Cells were washed twice, incubated with recombinant proteins, and stained as described above for subsequent analysis.

For transient surface protein expression, 2 × 10^5^ cells were seeded on 12-well plates and transiently transfected with 2 μg of plasmids encoding SARS-CoV-2 N (Addgene, no. 141391) or eGFP (Addgene, no. 141395) with TransIT-LT1. At indicated time after transfection, cells were processed as described above for cell surface protein binding assays.

For every assay and condition, at least 30,000 cells were analyzed on an BD FACSCelesta Cell Analyzer (BD Biosciences) with a high-throughput system unit, and quadrants in double staining plots were set on the basis of mock-infected condition for each cell type. Data were analyzed with FlowJo (Tree Star) and plotted with Prism v9.1.1 software (GraphPad).

### N protein transfer assays

For infectable and noninfectable cell coculture assays, 9 × 10^5^ cells of each indicated SARS-CoV-2–noninfectable cell type were stained with CellTrace Violet (Thermo Fisher Scientific, no. C34557), following the manufacturer’s instructions, and seeded in six-well plates. Then, 1 × 10^5^ CHO-K1_hACE2 cells (SARS-CoV-2–infectable) were homogeneously mixed and coseeded with indicated noninfectable cell type, being cocultured overnight. Cocultured cells were inoculated with SARS-CoV-2 at an MOI of 1 for 1 hour at 37°C, followed by removal of the virus inoculum and replacement of the medium containing 2% FBS. After 24 hours, cells were washed with DPBS and directly stained live on their six wells with Alexa Fluor–conjugated Abs and the LIVE/DEAD Fixable Violet Dead Cell Stain Kit, in DPBS for 25 min at 4°C. After staining, cells were washed twice with HBSS 0.1% BSA, trypsinized with TrypLE Express Enzyme for 5 min at 37°C, transferred to 96 wells, washed with HBSS 0.1% BSA, and fixed in 4% PFA for 30 min at room temperature. PFA was aspirated, and cells were resuspended in HBSS 0.1% BSA for flow cytometry analysis.

For transfected and nontransfected cell coculture assays, 2 × 10^5^ cells were seeded on six-well plates and transiently transfected with 2 μg of plasmids encoding SARS-CoV-2 N or eGFP with TransIT-LT1. After 24 hours, 9 × 10^5^ nontransfected cells were stained with CellTrace Violet and coseeded with their transfected homologs, being cocultured for 24 hours. Then, cells were washed with DPBS and directly stained live on their six wells with Alexa Fluor–conjugated Abs and the LIVE/DEAD Fixable Violet Dead Cell Stain Kit, in DPBS for 25 min at 4°C. Cells were washed twice with HBSS 0.1% BSA, trypsinized with TrypLE Express Enzyme for 5 min at 37°C, transferred to 96 wells, washed twice with HBSS 0.1% BSA, and resuspended in HBSS 0.1% BSA for flow cytometry analysis.

For transwell assays, 1 × 10^5^ CHO-K1 cells were stained with CellTrace Violet and seeded in 24-well plates (lower chamber). Then, 3 × 10^4^ Vero cells were plated inside 24-well transwell inserts (upper chamber, fig. S9) of 3-μm pore size (Corning, no. 351183). Cells on the upper chamber were inoculated with SARS-CoV-2 at an MOI of 1 for 1 hour at 37°C, as described above. After 24 hours, cells on each chamber were washed, trypsinized, transferred to 96 wells, washed, and stained with Alexa Fluor–conjugated Abs and the LIVE/DEAD Fixable Violet Dead Cell Stain Kit, in DPBS for 25 min at 4°C. After staining, cells were washed twice with HBSS 0.1% BSA and fixed in 4% PFA for 30 min at room temperature. PFA was aspirated, and cells were resuspended in HBSS 0.1% BSA for flow cytometry analysis. For every assay and condition, at least 100,000 (coculture assays) or 30,000 cells (transwell assays) were analyzed on a BD FACSCelesta Cell Analyzer with a high-throughput system unit. Data were analyzed with FlowJo and plotted with GraphPad Prism software.

### Cytokines and GAGs

Recombinant human cytokines used in this study [CCL1, CCL2, CCL3, CCL3L1, CCL4, CCL4L1, CCL5, CCL7, CCL8, CCL11, CCL13, CCL14, CCL15, CCL16, CCL17, CCL18, CCL19, CCL20, CCL21, CCL22, CCL23, CCL24, CCL25, CCL26, CCL27, CCL28, CXCL1, CXCL2, CXCL3, CXCL4, CXCL5, CXCL6, CXCL7, CXCL8, CXCL9, CXCL10, CXCL11, CXCL12α, CXCL12β, CXCL13, CXCL14, CXCL16, XCL1, CX3CL1, interleukin-1α (IL-1α), IL-1β, IL-6, IL-6Rα, IL-10, IL-12p70, IL-13, IL-17a, IL-18BP-Fc, IL-23, IL-27, IL-35, tumor necrosis factor–α (TNF-α), TNF-β, interferon-β (IFN-β), IFN-γ, IFN-λ1, and IFN-ω] from PeproTech and IFN-α2 and IL-18 from Sino Biological were reconstituted in DPBS 0.1% BSA at 10 μM, aliquoted, and stored at −80°C. Heparin (no. 2106), heparan sulfate from bovine kidney (no. H7640), chondroitin sulfate A (no. C9819), and chondroitin sulfate B (no. C3788) were obtained from MilliporeSigma. Heparan sulfate from porcine mucosa (no. AMS.GAG-HS01) and keratan sulfate (no. AMS.CSR-NAKPS2-SHC-1) were purchased from AMSBIO. We assumed an average molecular weight of 30 kDa for heparan sulfate from porcine mucosa and 15 kDa for heparin (*37*).

### BLI assays

High-throughput screening binding assays were performed on an Octet RED384 (ForteBio) instrument at 30°C with shaking at 1000 rpm. Streptavidin (SA) biosensors (Sartorius, no. 18-5019) were hydrated for 15 min in kinetics buffer (DPBS, 1% BSA, and 0.05% Tween 20). SARS-CoV-2 structural proteins and accessory factors (2X-StrepTag tagged) in lysis buffer from commercial sources or crude lysates of transfected cells (see details below) were loaded into SA biosensors up to 0.5 to 5 nm of binding response for 300 to 600 s, before baseline equilibration for 180 s in kinetics buffer. Association of each analyte in kinetics buffer at indicated concentration was carried out for 300 s, followed by dissociation for 300 s or longer. Standard binding and kinetic assays between SARS-CoV-2 N and GAGs or chemokines were performed as described above for binding assays. The negative signal of N binding to GAGs, expected given the large size of heparin molecules, was flipped prior further analysis (*18*, *38*). The data were baseline-subtracted before fitting performed using the homogeneous (1:1) and heterogeneous binding models (2:1; mass transport, 1:2) within the ForteBio Data Analysis HT software v12.0.1.55. Mean *K*_D_ (affinity constant), *k*_on_ (association rate constant), and *k*_off_ (dissociation rate constant) values were determined with a global fit applied to all data. The performance of each binding model fitting to the data was assessed on the basis of the lowest sum of the squared deviations or measure of error between the experimental data and the fitted line (χ^2^) and the highest correlation between fit and experimental data (*R*^2^).

The experiments were repeated with at least three independently produced batches of recombinant protein in crude lysates, obtained from 30 × 10^6^ HEK293-FT cells transfected with 30 μg of plasmids encoding SARS-CoV-2 structural proteins and accessory factors with TransIT-LT1. SARS-CoV-2 St containing 2X-StrepTag at the C-terminal region was commercially synthesized as mentioned above (GenScript Biotech). SARS-CoV-2 N, M, E, ORF3a, ORF3b, ORF6, ORF7a, ORF7b, ORF8, ORF9b, ORF9c, and ORF10 plasmids without signal peptide for secretion, described in (*39*), were acquired from Addgene (www.addgene.org/Nevan_Krogan). After 24 hours, transfected cells were selected with puromycin (10 μg/ml; InvivoGen, no. ant-pr-1). After 48 hours, transfected cells were trypsinized, washed with DPBS, and lysated for 30 min at 4°C in 1 ml of lysis buffer [50 mM tris-HCl (pH 7.4), 150 mM NaCl, 5 mM KCl, 5 mM MgCl_2_, 1% NP-40, and 1× protease inhibitors (Roche, no. 4693159001)], followed by centrifugation at 1000*g* at 4°C. Clarified supernatants (crude lysates) were collected, aliquoted, stored at −20°C, and characterized by immunoblotting (figs. S11B and S14), using mouse anti–2xStrep tag (1:1000; Qiagen, no. 34850) and secondary goat anti-mouse IgG IRDye 800CW (1:10,000; LI-COR, no. 926-32210). rN was additionally characterized by immunoblotting using IRDye 680RD SA (LI-COR, no. 926-68079) and human anti–SARS-CoV-2 N mAb (N18), followed by IRDye 680RD goat anti-human IgG secondary Ab (LI-COR, no. 926-68078).

For GAG competition assays of chemokine binding, selected chemokines (100 nM) were incubated in kinetics buffer alone or with indicated concentrations of soluble heparan sulfate from bovine kidney, chondroitin sulfate A and chondroitin sulfate B for 10 min at room temperature. The mixture was used to measure the association of N (in nanometers of binding response), as described above for the BLS binding assays. The value in nanometers of binding response of each chemokine binding without GAGs was considered 100%.

### Chemotaxis assays

Recombinant human CXCL12β (3 nM), alone or in combination with purified recombinant proteins, was placed in the lower chamber of a 96-well ChemoTx System plate (Neuro Probe, nos. 101-3 and 101-5) in RPMI 1640 containing 1% FBS. As internal controls within each assay, medium or recombinant protein alone was used. PBMCs, MonoMac-1, and MOLT-4 cells (1.25 × 10^5^) were placed on the upper compartment and separated from the lower chamber by a 3- or 5-μm pore size filter. The cells were incubated at 37°C for 3 hours in a humidified incubator with 5% CO_2_. The migrated cells in the lower chamber were stained with 5 μl of CellTiter 96 AQueous One Solution Cell Proliferation Assay (Promega, no. G3580) for 2 hours at 37°C with 5% CO_2_, measuring absorbance at 490 nm using a Synergy H1 plate reader (Bio-Tek).

The following recombinant proteins were used: SARS-CoV-2 S1-His (Sino Biological, no. 40591-V08B1), SARS-CoV-2 N-His (Sino Biological, no. 40588-V08B), SARS-CoV-2 N-His (Acro Biosystems, no. NUN-C5227), SARS-CoV-2 N-His (Ray Biotech, no. 230-30164), SARS-CoV-1 N-His (Acro Biosystems, no. NUN-S5229), and MERS-CoV N-His (Acro Biosystems, no. NUN-M52H5). SARS-CoV-2 N-His from Sino Biological (no. 40588-V08B) was used in all assays unless indicated.

### ADCC reporter assay

For each indicated cell type, 1 × 10^4^ cells were seeded on 96-well flat white tissue culture-treated plates (Thermo Fisher Scientific, no. 136101), cultured overnight, and infected with SARS-CoV-2 at an MOI of 1 (target cells). At 24 hpi, infected target cells were washed with DPBS, and the medium was replaced with 50 μl of RPMI 1640 with 4% low IgG serum (Promega, no. G711A) containing 5 × 10^4^ Jurkat effector cells (Promega, no. G701A) and serial dilutions of indicated human mAbs. After overnight incubation at 37°C with 5% CO_2_, 50 μl of Bright-Glo Luciferase Assay lysis/substrate buffer (Promega, no. E2620) was added, and luminescence was measured after 10 min using a POLARstar Omega plate reader (BMG LABTECH) within the luciferase glow assay template and the following parameters: gain, 3600; measurement interval time, 0.1 s; and maximum counts, 2 × 10^6^. Measurements were performed in triplicate and relative luciferase units were plotted and analyzed with GraphPad Prism software. Data fitting with GraphPad Prism was performed with the nonlinear regression dose-response stimulation model.

### Statistical analysis

Statistical analyses were performed using GraphPad Prism software. When indicated, *P* values were calculated using Student’s two-tailed unpaired *t* test (at 99% confidence interval), and *P* < 0.01 was considered statistically significant. On the other hand, one-way analysis of variance (ANOVA) and Dunnett’s multiple comparisons test (at 95% confidence interval) were used to compare all conditions against untreated or mock-infected cells (as indicated for each case), considering *P* < 0.05 as statistically significant.

## Supplementary Materials

### Other Supplementary Material for this manuscript includes the following:

Movies S1 to S14

Animations S1 to S14

## Notes

View/request a protocol for this paper from *Bio-protocol*.

## References

[1] Q.-X. Long, B.-Z. Liu, H.-J. Deng, G.-C. Wu, K. Deng, Y.-K. Chen, P. Liao, J.-F. Qiu, Y. Lin, X.-F. Cai, D.-Q. Wang, Y. Hu, J.-H. Ren, N. Tang, Y.-Y. Xu, L.-H. Yu, Z. Mo, F. Gong, X.-L. Zhang, W.-G. Tian, L. Hu, X.-X. Zhang, J.-L. Xiang, H.-X. Du, H.-W. Liu, C.-H. Lang, X.-H. Luo, S.-B. Wu, X.-P. Cui, Z. Zhou, M.-M. Zhu, J. Wang, C.-J. Xue, X.-F. Li, L. Wang, Z.-J. Li, K. Wang, C.-C. Niu, Q.-J. Yang, X.-J. Tang, Y. Zhang, X.-M. Liu, J.-J. Li, D.-C. Zhang, F. Zhang, P. Liu, J. Yuan, Q. Li, J.-L. Hu, J. Chen, A.-L. Huang, Antibody responses to SARS-CoV-2 in patients with COVID-19. Nat. Med. 26, 845–848 (2020). 32350462 10.1038/s41591-020-0897-1

[2] A. Sariol, S. Perlman, Lessons for COVID-19 immunity from other coronavirus infections. Immunity 53, 248–263 (2020). 32717182 10.1016/j.immuni.2020.07.005 PMC7359787

[3] J. W. Yewdell, E. Frank, W. Gerhard, Expression of influenza A virus internal antigens on the surface of infected P815 cells. J. Immunol. 126, 1814–1819 (1981). 7217668

[4] J. W. Yewdell, J. R. Bennink, M. Mackett, L. Lefrancois, D. S. Lyles, B. Moss, Recognition of cloned vesicular stomatitis virus internal and external gene products by cytotoxic T lymphocytes. J. Exp. Med. 163, 1529–1538 (1986). 3011949 10.1084/jem.163.6.1529 PMC2188125

[5] U. D. Staerz, J. W. Yewdell, M. J. Bevan, Hybrid antibody-mediated lysis of virus-infected cells. Eur. J. Immunol. 17, 571–574 (1987). 3494618 10.1002/eji.1830170422

[6] M. W. Lamere, H. T. Lam, A. Moquin, L. Haynes, F. E. Lund, T. D. Randall, D. A. Kaminski, Contributions of antinucleoprotein IgG to heterosubtypic immunity against influenza virus. J. Immunol. 186, 4331–4339 (2011). 21357542 10.4049/jimmunol.1003057 PMC3159153

[7] J. C. Marie, F. Saltel, J. M. Escola, P. Jurdic, T. F. Wild, B. Horvat, Cell surface delivery of the measles virus nucleoprotein: A viral strategy to induce immunosuppression. J. Virol. 78, 11952–11961 (2004). 15479835 10.1128/JVI.78.21.11952-11961.2004 PMC523264

[8] P. F. Céspedes, S. M. Bueno, B. A. Ramírez, R. S. Gomez, S. A. Riquelme, C. E. Palavecino, J. P. Mackern-Oberti, J. E. Mora, D. Depoil, C. Sacristán, M. Cammer, A. Creneguy, T. H. Nguyen, C. A. Riedel, M. L. Dustin, A. M. Kalergis, Surface expression of the hRSV nucleoprotein impairs immunological synapse formation with T cells. Proc. Natl. Acad. Sci. U.S.A. 111, E3214–E3223 (2014). 25056968 10.1073/pnas.1400760111 PMC4128097

[9] T. Straub, O. Schweier, M. Bruns, F. Nimmerjahn, A. Waisman, H. Pircher, Nucleoprotein-specific nonneutralizing antibodies speed up LCMV elimination independently of complement and FcγR. Eur. J. Immunol. 43, 2338–2348 (2013). 23749409 10.1002/eji.201343565

[10] K. Ikuta, C. Morita, S. Miyake, T. Ito, M. Okabayashi, K. Sano, M. Nakai, K. Hirai, S. Kato, Expression of human immunodeficiency virus type 1 (HIV-1) gag antigens on the surface of a cell line persistently infected with HIV-1 that highly expresses HIV-1 antigens. Virology 170, 408–417 (1989). 2499113 10.1016/0042-6822(89)90431-5

[11] J. Buchrieser, J. Dufloo, M. Hubert, B. Monel, D. Planas, M. M. Rajah, C. Planchais, F. Porrot, F. Guivel-Benhassine, S. Van der Werf, N. Casartelli, H. Mouquet, T. Bruel, O. Schwartz, Syncytia formation by SARS-CoV-2-infected cells. EMBO J. 39, e106267 (2020). 33051876 10.15252/embj.2020106267 PMC7646020

[12] H. Van Phan, M. van Gent, N. Drayman, A. Basu, M. U. Gack, S. Tay, Fixed single-cell RNA sequencing for understanding virus infection and host response (2021); 10.1101/2020.09.17.302232v2. PMC8463713 34561439

[13] B. Boson, V. Legros, B. Zhou, E. Siret, C. Mathieu, F. L. Cosset, D. Lavillette, S. Denolly, The SARS-CoV-2 envelope and membrane proteins modulate maturation and retention of the spike protein, allowing assembly of virus-like particles. J. Biol. Chem. 296, 100111 (2021). 33229438 10.1074/jbc.RA120.016175 PMC7833635

[14] D. C. Dinesh, D. Chalupska, J. Silhan, E. Koutna, R. Nencka, V. Veverka, E. Boura, Structural basis of RNA recognition by the SARS-CoV-2 nucleocapsid phosphoprotein. PLOS Pathog. 16, e1009100 (2020). 33264373 10.1371/journal.ppat.1009100 PMC7735635

[15] L. Zinzula, J. Basquin, S. Bohn, F. Beck, S. Klumpe, G. Pfeifer, I. Nagy, A. Bracher, F. U. Hartl, W. Baumeister, High-resolution structure and biophysical characterization of the nucleocapsid phosphoprotein dimerization domain from the Covid-19 severe acute respiratory syndrome coronavirus 2. Biochem. Biophys. Res. Commun. 538, 54–62 (2021). 33039147 10.1016/j.bbrc.2020.09.131 PMC7532810

[16] I. Capila, R. J. Linhardt, Heparin-protein interactions. Angew. Chem. Int. Ed. Engl. 41, 391–412 (2002). 12491369 10.1002/1521-3773(20020201)41:3<390::aid-anie390>3.0.co;2-b

[17] P. Hossler, S. F. Khattak, Z. J. Li, Optimal and consistent protein glycosylation in mammalian cell culture. Glycobiology 19, 936–949 (2009). 19494347 10.1093/glycob/cwp079

[18] Sartorius Lab Instruments GmbH & Co., “Biomolecular Binding Kinetics Assays on the Octet® Platform” (2021); www.sartorius.com/resource/blob/742330/05671fe2de45d16bd72b8078ac28980d/octet-biomolecular-binding-kinetics-application-note-4014-en-1--data.pdf [the easiest access to this source is via the URL].

[19] K. Nakanaga, K. Yamanouchi, K. Fujiwara, Protective effect of monoclonal antibodies on lethal mouse hepatitis virus infection in mice. J. Virol. 59, 168–171 (1986). 3012115 10.1128/jvi.59.1.168-171.1986 PMC253053

[20] J. Lecomte, V. Cainelli-Gebara, G. Mercier, S. Mansour, P. J. Talbot, G. Lussier, D. Oth, Protection from mouse hepatitis virus type 3-induced acute disease by an anti-nucleoprotein monoclonal antibody. Brief report. Arch. Virol. 97, 123–130 (1987). 2825619 10.1007/BF01310740 PMC7086664

[21] N. T. Supekar, A. Shajahan, A. S. Gleinich, D. S. Rouhani, C. Heiss, D. G. Chapla, K. W. Moremen, P. Azadi, Variable posttranslational modifications of severe acute respiratory syndrome coronavirus 2 nucleocapsid protein. Glycobiology 9, 1080–1092 (2021). 10.1093/glycob/cwab044 PMC8241430 33997890

[22] C. Rabouille, Pathways of unconventional protein secretion. Trends Cell Biol. 27, 230–240 (2017). 27989656 10.1016/j.tcb.2016.11.007

[23] T. Katsinelos, M. Zeitler, E. Dimou, A. Karakatsani, H. M. Müller, E. Nachman, J. P. Steringer, C. Ruiz de Almodovar, W. Nickel, T. R. Jahn, Unconventional secretion mediates the trans-cellular spreading of Tau. Cell Rep. 23, 2039–2055 (2018). 29768203 10.1016/j.celrep.2018.04.056

[24] V. Gonzalez-Motos, K. A. Kropp, A. Viejo-Borbolla, Chemokine binding proteins: An immunomodulatory strategy going viral. Cytokine Growth Factor Rev. 30, 71–80 (2016). 26987612 10.1016/j.cytogfr.2016.02.007

[25] B. Hernaez, A. Alcamí, Virus-encoded cytokine and chemokine decoy receptors. Curr. Opin. Immunol. 66, 50–56 (2020). 32408109 10.1016/j.coi.2020.04.008

[26] C. Gaebler, Z. Wang, J. C. C. Lorenzi, F. Muecksch, S. Finkin, M. Tokuyama, A. Cho, M. Jankovic, D. Schaefer-Babajew, T. Y. Oliveira, M. Cipolla, C. Viant, C. O. Barnes, Y. Bram, G. Breton, T. Hägglöf, P. Mendoza, A. Hurley, M. Turroja, K. Gordon, K. G. Millard, V. Ramos, F. Schmidt, Y. Weisblum, D. Jha, M. Tankelevich, G. Martinez-Delgado, J. Yee, R. Patel, J. Dizon, C. Unson-O’Brien, I. Shimeliovich, D. F. Robbiani, Z. Zhao, A. Gazumyan, R. E. Schwartz, T. Hatziioannou, P. J. Bjorkman, S. Mehandru, P. D. Bieniasz, M. Caskey, M. C. Nussenzweig, Evolution of antibody immunity to SARS-CoV-2. Nature 591, 639–644 (2021). 33461210 10.1038/s41586-021-03207-w PMC8221082

[27] A. E. Grootemaat, S. van der Niet, E. R. Scholl, E. Roos, B. Schurink, M. Bugiani, S. E. Miller, P. Larsen, J. Pankras, E. A. Reits, N. N. van der Wel, Lipid and nucleocapsid N-protein accumulation in COVID-19 patient lung and infected cells. Microbiol Spectr. 10, e0127121 (2022). 35171025 10.1128/spectrum.01271-21 PMC8849100

[28] S.-J. Liu, C.-H. Leng, S.-P. Lien, H.-Y. Chi, C.-Y. Huang, C.-L. Lin, W.-C. Lian, C.-J. Chen, S.-L. Hsieh, P. Chong, Immunological characterizations of the nucleocapsid protein based SARS vaccine candidates. Vaccine 24, 3100–3108 (2006). 16494977 10.1016/j.vaccine.2006.01.058 PMC7115648

[29] T. Dangi, J. Class, N. Palacio, J. M. Richner, P. Penaloza MacMaster, Combining spike- and nucleocapsid-based vaccines improves distal control of SARS-CoV-2. Cell Rep. 36, 109664 (2021). 34450033 10.1016/j.celrep.2021.109664 PMC8367759

[30] V. Joag, S. Wijeyesinghe, J. M. Stolley, C. F. Quarnstrom, T. Dileepan, A. G. Soerens, J. A. Sangala, S. D. O’Flanagan, N. V. Gavil, S. W. Hong, S. Bhela, S. Gangadhara, E. Weyu, W. E. Matchett, J. Thiede, V. Krishna, M. C. Cheeran, T. D. Bold, R. Amara, P. Southern, G. T. Hart, L. Schifanella, V. Vezys, M. K. Jenkins, R. A. Langlois, D. Masopust, Cutting edge: Mouse SARS-CoV-2 epitope reveals infection and vaccine-elicited CD8 T cell responses. J. Immunol. 206, 931–935 (2021). 33441437 10.4049/jimmunol.2001400 PMC8136468

[31] W. E. Matchett, V. Joag, J. M. Stolley, F. K. Shepherd, C. F. Quarnstrom, C. K. Mickelson, S. Wijeyesinghe, A. G. Soerens, S. Becker, J. M. Thiede, E. Weyu, S. D. O’Flanagan, J. A. Walter, M. N. Vu, V. D. Menachery, T. D. Bold, V. Vezys, M. K. Jenkins, R. A. Langlois, D. Masopust, Cutting edge: Nucleocapsid vaccine elicits spike-independent SARS-CoV-2 protective immunity. J. Immunol. 207, 376–379 (2021). 34193597 10.4049/jimmunol.2100421 PMC8516699

[32] I. Sagara, A. Dicko, A. Zeguime, M’Bouye Doucoure, J. Kwan, I. Zaidi, J. Doritchamou, M. Snow-Smith, N. Alani, J. Renn, I. Kosik, J. Holly, J. Yewdell, D. Esposito, K. Sadtler, P. Duffy, SARS-CoV-2 seroassay optimization and performance in a population with high background reactivity in Mali (2021); 10.1101/2021.03.08.21252784v1. PMC8522418 34612499

[33] E. Kowarz, D. Löscher, R. Marschalek, Optimized Sleeping Beauty transposons rapidly generate stable transgenic cell lines. Biotechnol. J. 10, 647–653 (2015). 25650551 10.1002/biot.201400821

[34] A. Zhao, W. Qin, Y. Han, W. Wen, W. Zhang, Z. Lian, G. Chen, Z. Zhang, J. Peng, H. Wang, Y. Guo, Isolation and identification of an scFv antibody against nucleocapsid protein of SARS-CoV. Microbes Infect. 9, 1026–1033 (2007). 17548223 10.1016/j.micinf.2007.04.008 PMC7110486

[35] Y. Wu, F. Wang, C. Shen, W. Peng, D. Li, C. Zhao, Z. Li, S. Li, Y. Bi, Y. Yang, Y. Gong, H. Xiao, Z. Fan, S. Tan, G. Wu, W. Tan, X. Lu, C. Fan, Q. Wang, Y. Liu, C. Zhang, J. Qi, G. F. Gao, F. Gao, L. Liu, A noncompeting pair of human neutralizing antibodies block COVID-19 virus binding to its receptor ACE2. Science 368, 1274–1278 (2020). 32404477 10.1126/science.abc2241 PMC7223722

[36] D. Wrapp, N. Wang, K. S. Corbett, J. A. Goldsmith, C. L. Hsieh, O. Abiona, B. S. Graham, J. S. McLellan, Cryo-EM structure of the 2019-nCoV spike in the prefusion conformation. Science 367, 1260–1263 (2020). 32075877 10.1126/science.abb2507 PMC7164637

[37] S. Schulman, M. Levi, Antithrombotic Therapy, in *Williams Hematology 10th Edition* (McGraw Hill, 2021), chap. 32.

[38] Sartorius Lab Instruments GmbH & Co., “Octet® Potency Assay: Development, Qualification and Validation Strategies” (2021); www.sartorius.com/download/789382/octet-potency-assay-development-validation-strategies-applic-2--data.pdf [the easiest access to this source is via the URL].

[39] D. E. Gordon, G. M. Jang, M. Bouhaddou, J. Xu, K. Obernier, K. M. White, M. J. O’Meara, V. V. Rezelj, J. Z. Guo, D. L. Swaney, T. A. Tummino, R. Hüttenhain, R. M. Kaake, A. L. Richards, B. Tutuncuoglu, H. Foussard, J. Batra, K. Haas, M. Modak, M. Kim, P. Haas, B. J. Polacco, H. Braberg, J. M. Fabius, M. Eckhardt, M. Soucheray, M. J. Bennett, M. Cakir, M. J. McGregor, Q. Li, B. Meyer, F. Roesch, T. Vallet, A. M. Kain, L. Miorin, E. Moreno, Z. Z. C. Naing, Y. Zhou, S. Peng, Y. Shi, Z. Zhang, W. Shen, I. T. Kirby, J. E. Melnyk, J. S. Chorba, K. Lou, S. A. Dai, I. Barrio-Hernandez, D. Memon, C. Hernandez-Armenta, J. Lyu, C. J. P. Mathy, T. Perica, K. B. Pilla, S. J. Ganesan, D. J. Saltzberg, R. Rakesh, X. Liu, S. B. Rosenthal, L. Calviello, S. Venkataramanan, J. Liboy-Lugo, Y. Lin, X. P. Huang, Y. Liu, S. A. Wankowicz, M. Bohn, M. Safari, F. S. Ugur, C. Koh, N. S. Savar, Q. D. Tran, D. Shengjuler, S. J. Fletcher, M. C. O’Neal, Y. Cai, J. C. J. Chang, D. J. Broadhurst, S. Klippsten, P. P. Sharp, N. A. Wenzell, D. Kuzuoglu-Ozturk, H. Y. Wang, R. Trenker, J. M. Young, D. A. Cavero, J. Hiatt, T. L. Roth, U. Rathore, A. Subramanian, J. Noack, M. Hubert, R. M. Stroud, A. D. Frankel, O. S. Rosenberg, K. A. Verba, D. A. Agard, M. Ott, M. Emerman, N. Jura, M. von Zastrow, E. Verdin, A. Ashworth, O. Schwartz, C. d’Enfert, S. Mukherjee, M. Jacobson, H. S. Malik, D. G. Fujimori, T. Ideker, C. S. Craik, S. N. Floor, J. S. Fraser, J. D. Gross, A. Sali, B. L. Roth, D. Ruggero, J. Taunton, T. Kortemme, P. Beltrao, M. Vignuzzi, A. García-Sastre, K. M. Shokat, B. K. Shoichet, N. J. Krogan, A SARS-CoV-2 protein interaction map reveals targets for drug repurposing. Nature 583, 459–468 (2020). 32353859 10.1038/s41586-020-2286-9 PMC7431030

