# Supplementary material for: Cell surface SARS-CoV-2 nucleocapsid protein modulates innate and adaptive immunity
Source: Sci Adv. 2022 Aug 3;8(31):eabp9770. doi: 10.1126/sciadv.abp9770 (PMC9348789; doi:10.1126/sciadv.abp9770)
Supplement: Supplementary file 1 — Figs. S1 to S14 [file sciadv.abp9770_sm.pdf]

Science Advances
AAAS

Supplementary Materials for

Cell surface SARS-CoV-2 nucleocapsid protein modulates innate and adaptive immunity

Alberto Domingo López-Muñoz *et al.*

Corresponding author: Jonathan W. Yewdell, jyewdell@nih.gov

Sci. Adv. **8**, eabp9770 (2022)
DOI: 10.1126/sciadv.abp9770

The PDF file includes:

Figs. S1 to S14

Other Supplementary Material for this manuscript includes the following:

Movies S1 to S14
Animations S1 to S14

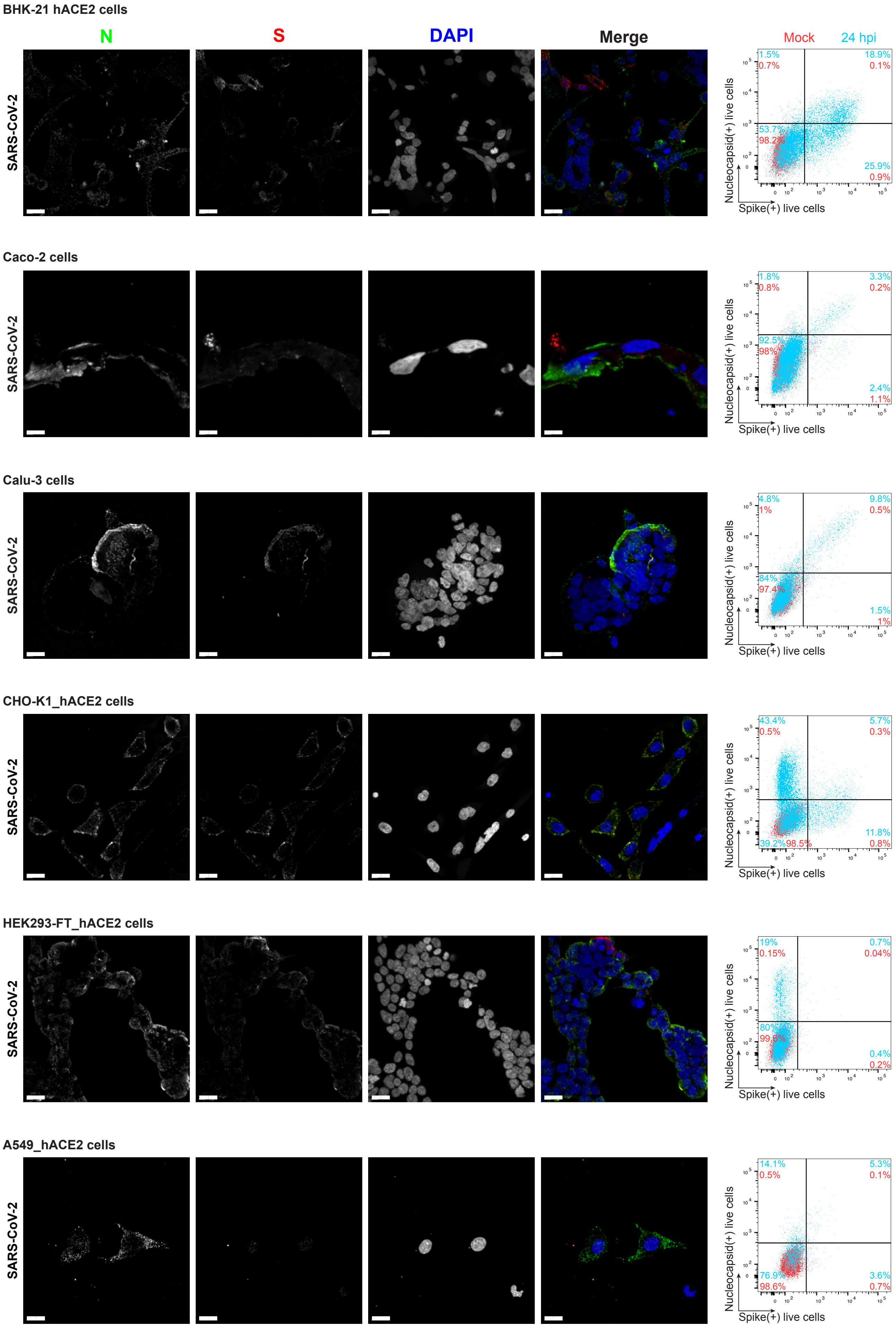

Fig. S1. SARS-CoV-2 N protein is expressed on the cell surface of diverse infected cell types. Maximum intensity projections (MIP) of laser confocal microscopy z-stack images and histogram overlays of flow cytometry analyses of live wild-type SARS-CoV-2-infected BHK-21_hACE2, Caco-2, Calu-3, CHO-K1_hACE2, HEK293-FT_hACE2 and A549_hACE2 cells stained live with Abs at 24 hpi (MOI = 1). Scale bars = 20 µm. Images are representative of three independent experiments with similar results. Representative plots of flow cytometry analyses show double staining of surface S and N, indicating the percentage of the gated cell population for each quadrant of the double staining. Data are representative of one experiment out of at least three independent experiments performed in triplicate.

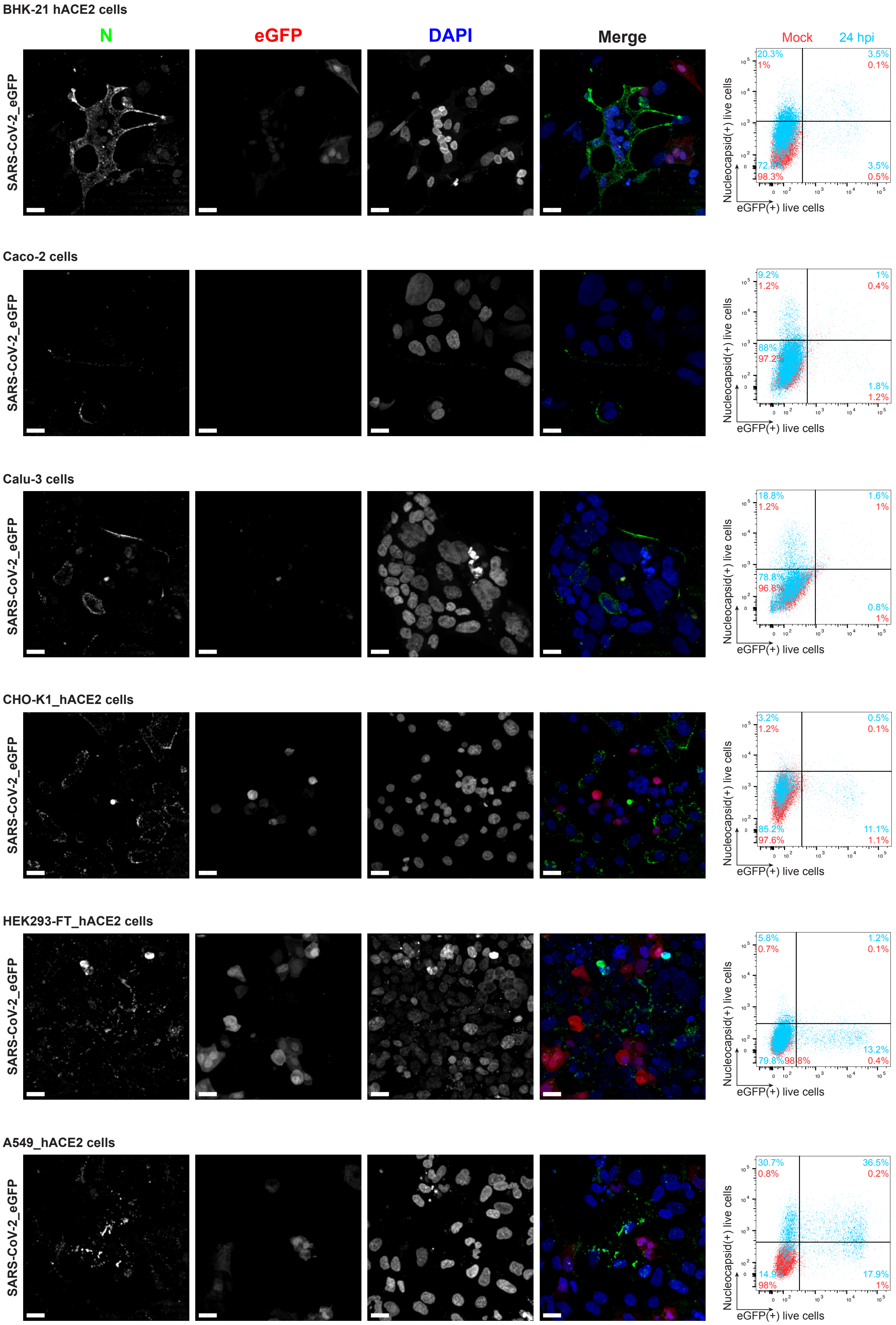

Fig. S2. SARS-CoV-2 N protein is a robust marker of cell-surface infection across cell types. MIP of laser confocal microscopy z-stack images and histogram overlays of flow cytometry analyses of live eGFP expressing SARS-CoV-2-infected BHK-21_hACE2, Caco-2, Calu-3, CHO-K1_hACE2, HEK293-FT_hACE2 and A549_hACE2 cells stained live with MAb against N at 24 hpi (MOI = 1). Scale bars = 20 µm. Images are representative of two independent experiments with similar results. Representative plots of flow cytometry analyses show double staining of eGFP and surface N, indicating the percentage of the gated cell population for each quadrant of the double staining. Data are representative of one experiment out of at least three independent experiments performed in triplicate.

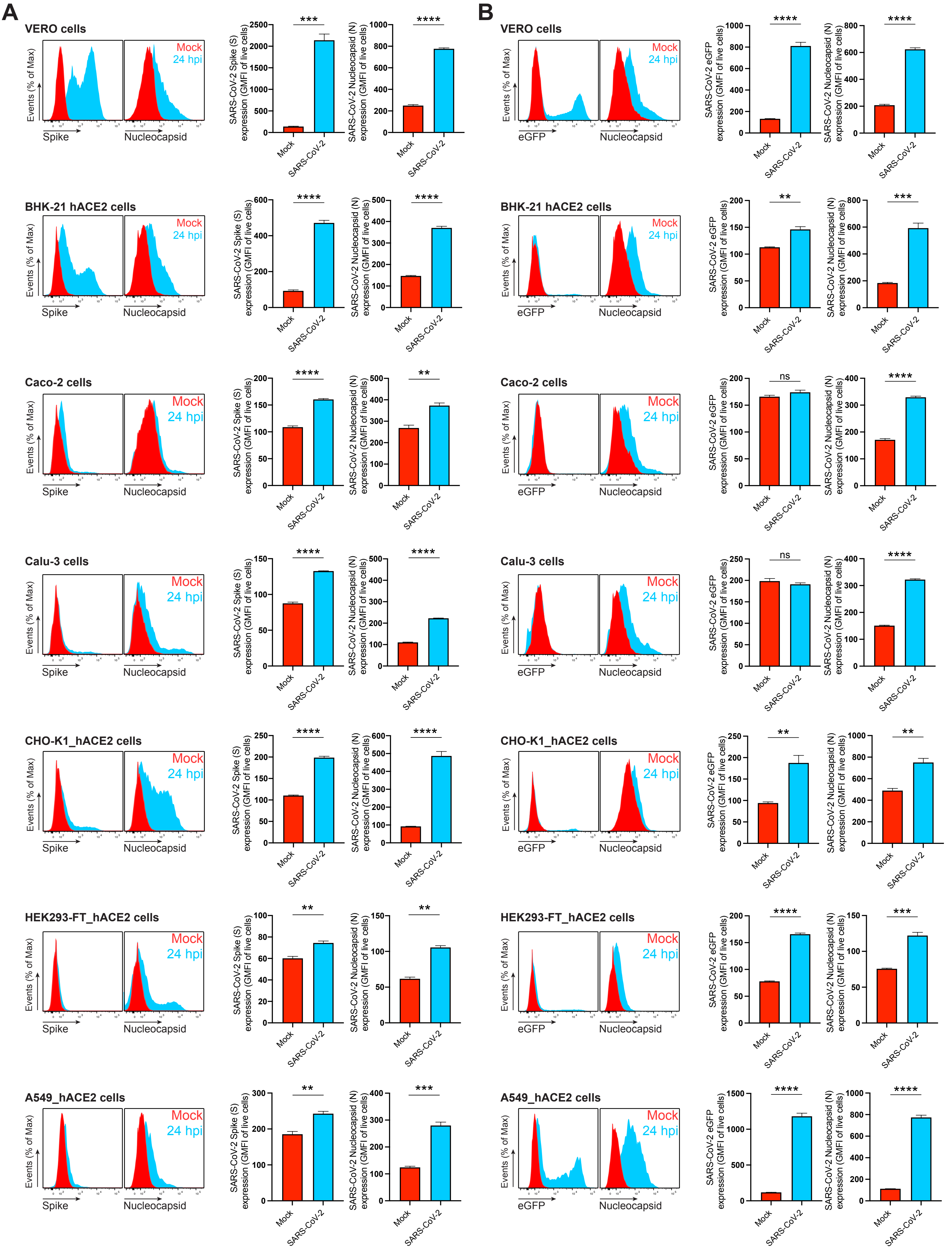

Fig. S3. SARS-CoV-2 N is significantly detected on the cell surface of all live infected cell types tested in this study. Flow cytometry analyses of Vero, BHK-21_hACE2, Caco-2, Calu-3, CHO-K1_hACE2, HEK293-FT_hACE2 and A549_hACE2 cells inoculated with wild-type (**A**) or eGFP reported (**B**) SARS-CoV-2 (MOI = 1), and stained live with Abs at 24 hpi against SARS-CoV-2 S and N proteins. For each cell type and infection, the following is shown: histogram overlays of surface S and N, and intracellular eGFP, as well as the geometric mean fluorescent intensity (GMFI) is plotted showing mean +/- SEM (n = 3). Histogram overlays correspond to **Fig. 1B** (Vero cells), while those others shown in (**A**) to **Fig. S1** and those in (**B**) to **Fig. S2**. Data are representative of one experiment out of at least three independent experiments performed in triplicate. *ns* (nonsignificant) $p > 0.01$, ** $p < 0.01$, *** $p < 0.001$, **** $p < 0.0001$ by Student s two-tailed unpaired *t*-test.

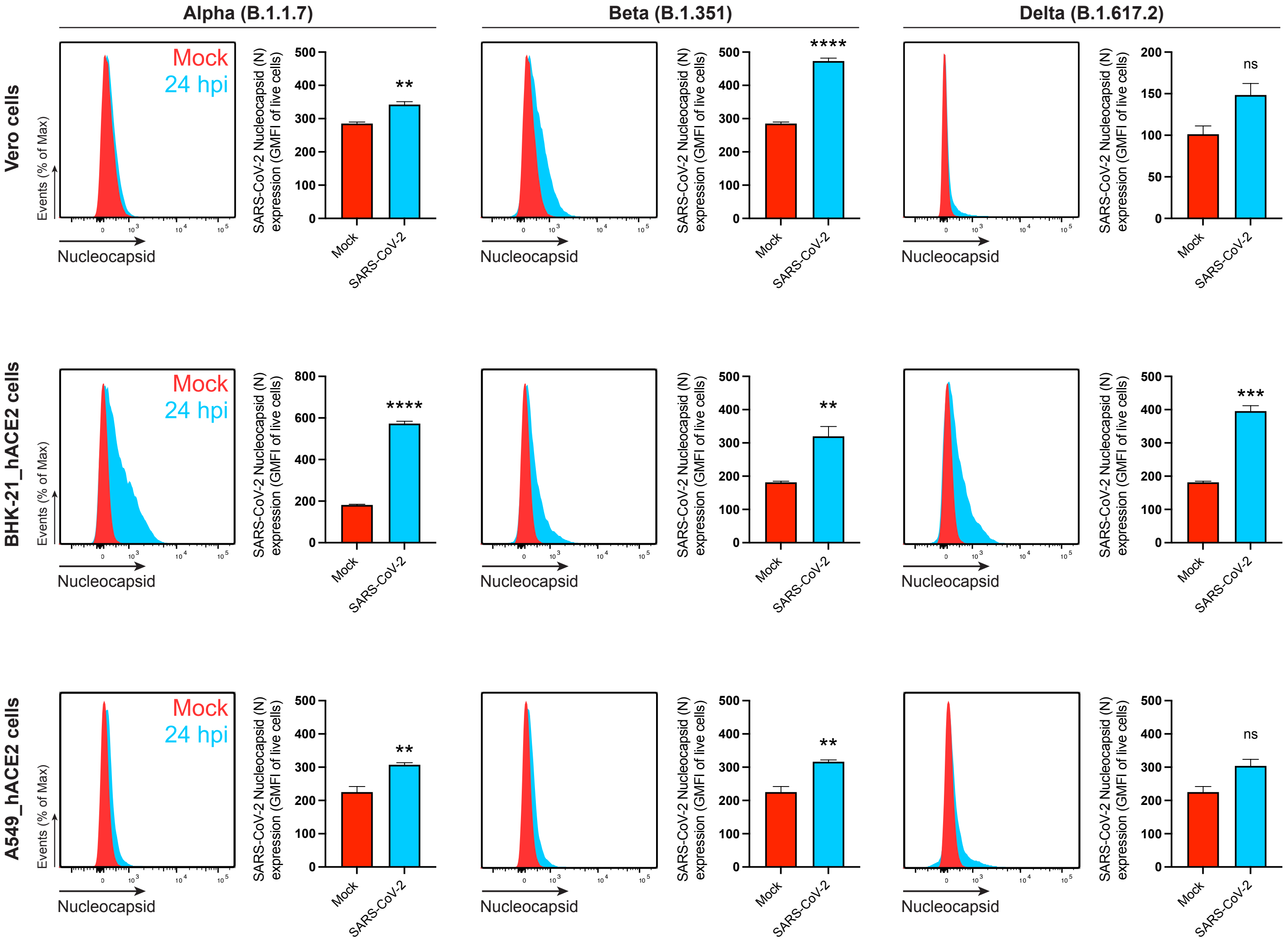

Fig. S4. SARS-CoV-2 N is also detected on the cell surface of live cells infected with the Alpha, Beta and Delta variants. Flow cytometry analyses of Vero, BHK-21_hACE2, and A549_hACE2 cells inoculated with SARS-CoV-2 variants (MOI = 1), and stained live with Abs at 24 hpi against SARS-CoV-2 N. For each cell type and infection, the following is shown: histogram overlays of surface N on live cells, as well as the GMFI is plotted showing mean +/- SEM (n = 3). Data are representative of one experiment out of at least three independent experiments performed in triplicate. *ns* (nonsignificant) $p > 0.01$, ** $p < 0.01$, *** $p < 0.001$, **** $p < 0.0001$ by Student s two-tailed unpaired *t*-test.

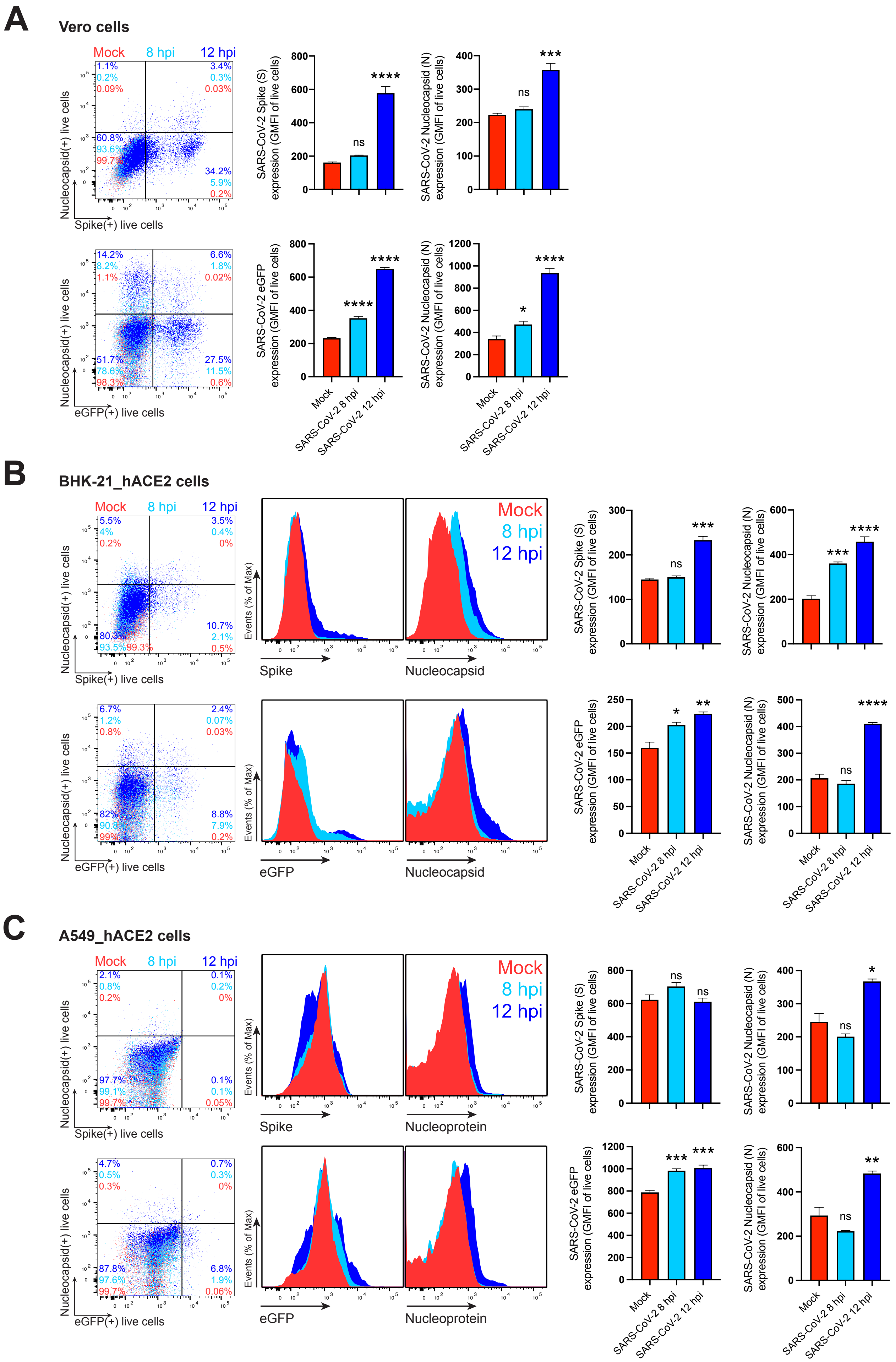

Fig. S5. SARS-CoV-2 N reaches the cell surface as soon as 8 to 12 hpi. Time course of surface S, N, and eGFP proteins expression in live Vero (**A**), BHK-21_hACE2 (**B**) and A549_hACE2 cells (**C**) infected with wild-type and eGFP reporter SARS-CoV-2 at 8 and 12 hpi (MOI = 1). Representative plots of flow cytometry analyses show double staining of surface S, N, and intracellular eGFP, indicating the percentage of the gated cell population for each quadrant. Histogram overlays of surface S and N, and intracellular eGFP are shown, as well as the GMFI is plotted showing mean +/- SEM (n = 3). Histogram overlays from Vero cells analyses are shown in **Fig. 1A**, **1B**. Data are representative of one experiment out of at least three independent experiments performed in triplicate. One-way ANOVA and Dunnett s Multiple comparison test were used to compare all conditions against mock-infected cells: *ns* (nonsignificant) $p > 0.05$, * $p < 0.05$, ** $p < 0.01$, *** $p < 0.001$, **** $p < 0.0001$.

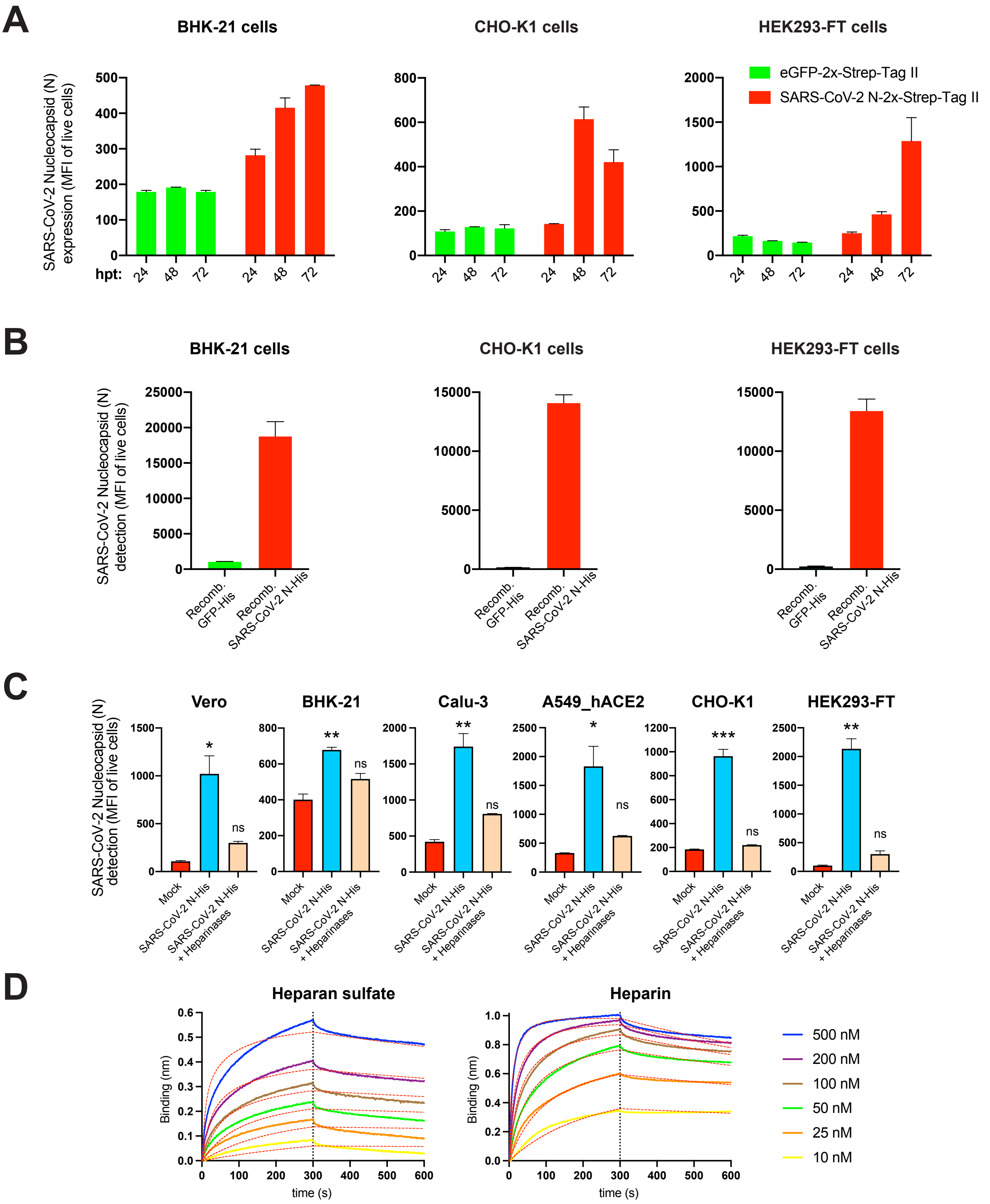

E

GAG	K_D (M)	k_{on} (1/M.s)	K_{off} (1/s)	R^2
Heparan sulfate	$8.9\times10^{-9} \pm 6.7\times10^{-10}$	$6.6\times10^{4} \pm 2.6\times10^{3}$	$5.9\times10^{-4} \pm 3.8\times10^{-5}$	0.977
Heparin	$4.4\times10^{-9} \pm 8.1\times10^{-11}$	$3.8\times10^{5} \pm 4.8\times10^{3}$	$1.6\times10^{-3} \pm 2.6\times10^{-5}$	0.983

Values reported represent the average of the global fit (± error) to the data from at least three independent BLI kinetic assays performed with different N protein preparations. Kinetics parameters were determined by using the bivalent analyte (1:2) binding model.

Fig. S6. Other SARS-CoV-2 genes are not required for N cell surface expression and binding to surface heparan sulfate/heparin. (**A**) Flow cytometry analyses of surface N kinetics (24-72 h) expression in BHK-21, CHO-K1 and HEK293-FT cells transiently transfected with a plasmid encoding eGFP or N protein, detected with Abs. (**B**) Flow cytometry analyses of exogenous rN binding to BHK-21, CHO-K1 and HEK293-FT cells, incubated with recombinant eGFP or N protein (100 ng) for 15 min, washed twice, and stained live with Abs. (**C**) Flow cytometry analyses of Vero, BHK-21, Calu-3, A549_hACE2, CHO-K1 and HEK293-FT cells treated with heparinases for 1 h, washed twice, incubated with 50 ng of rN protein for 15 min, washed twice again, stained live with Abs, and analyzed. The MFI of expressed (**A**) or bound (**B**, **C**) surface N protein from live cells is plotted for each case, showing mean +/- SEM (n = 2). In (**C**), One-way ANOVA and Dunnett s Multiple comparison test were used to compare all conditions against untreated cells (mock): *ns* (nonsignificant) $p > 0.05$, * $p < 0.05$, ** $p < 0.01$, *** $p < 0.001$. (**D**) BLI sensorgrams of kinetic assays depicting the interaction between immobilized N protein and different concentrations of heparan sulfate and heparin. All curves were analyzed with the ForteBio Data Analysis HT software, where red dashed lines correspond to a global fit of the data using the bivalent analyte model (1:2). (**E**) Table showing averaged values from the kinetic analyses of the N protein binding to heparan sulfate and heparin by BLI. All analyses were repeated with different protein preparations, and one representative assay out of at least three independent assays performed in duplicate is shown.

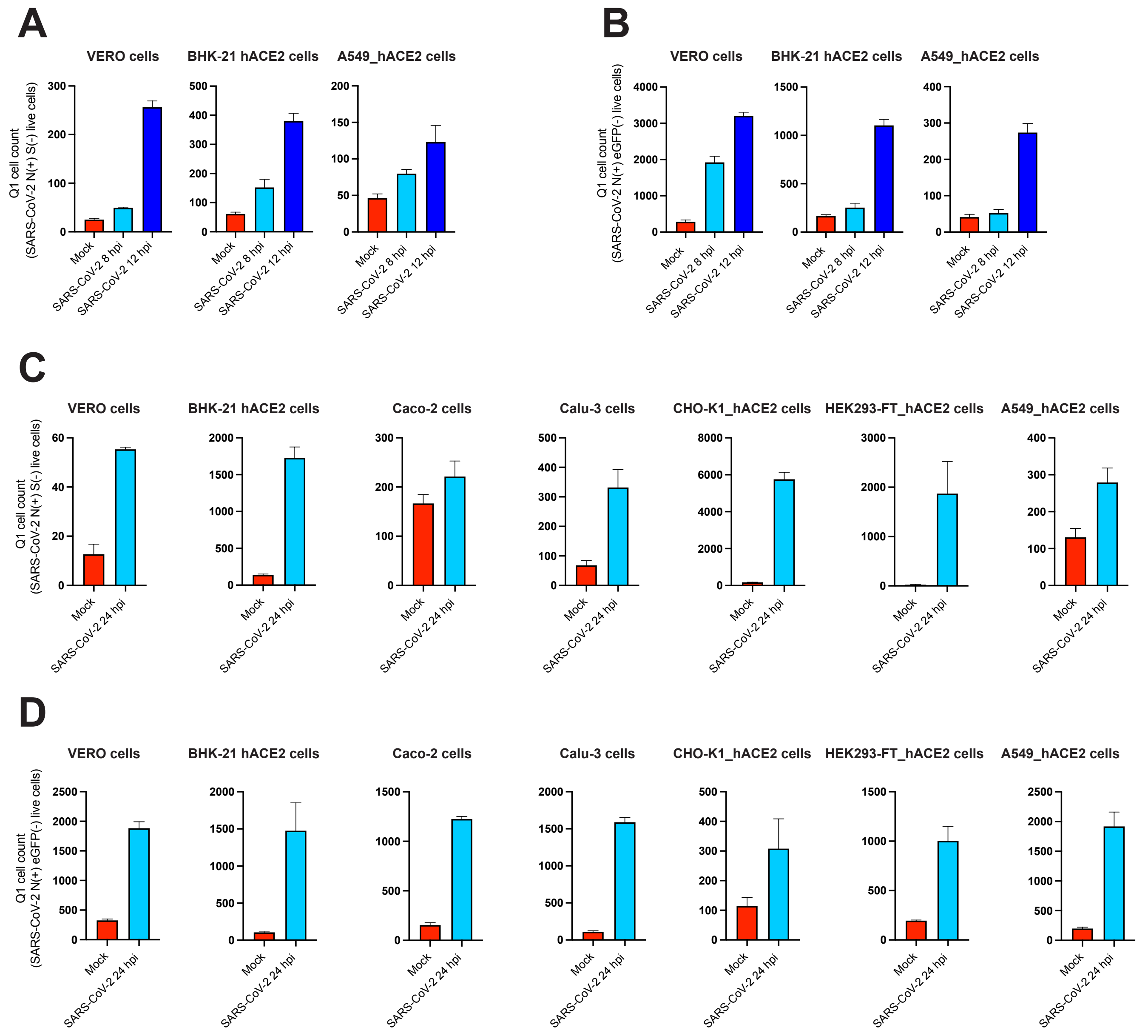

Fig. S7. Additional evidence supporting transfer of N from infected to uninfected infected cells. (**A-D**) Cells expressing N but no other marker of infection (S or eGFP) increase in a time-dependent manner. Quadrant 1 (Q1) in plots of flow cytometry analyses showing double staining of surface N and S/eGFP identifies a cell subset of cells only expressing N during infection. Bar histograms show mean +/- SEM (n = 3) from Q1 cell counts of cells at 8 and 12 h after infection with wild-type (**A**) or eGFP reporter virus (**B**), as well as at 24 hpi for each cell type analyzed after wild-type (**C**) or eGFP reporter virus (**D**) infection. Data are representative of one experiment out of at least three independent experiments performed in triplicate.

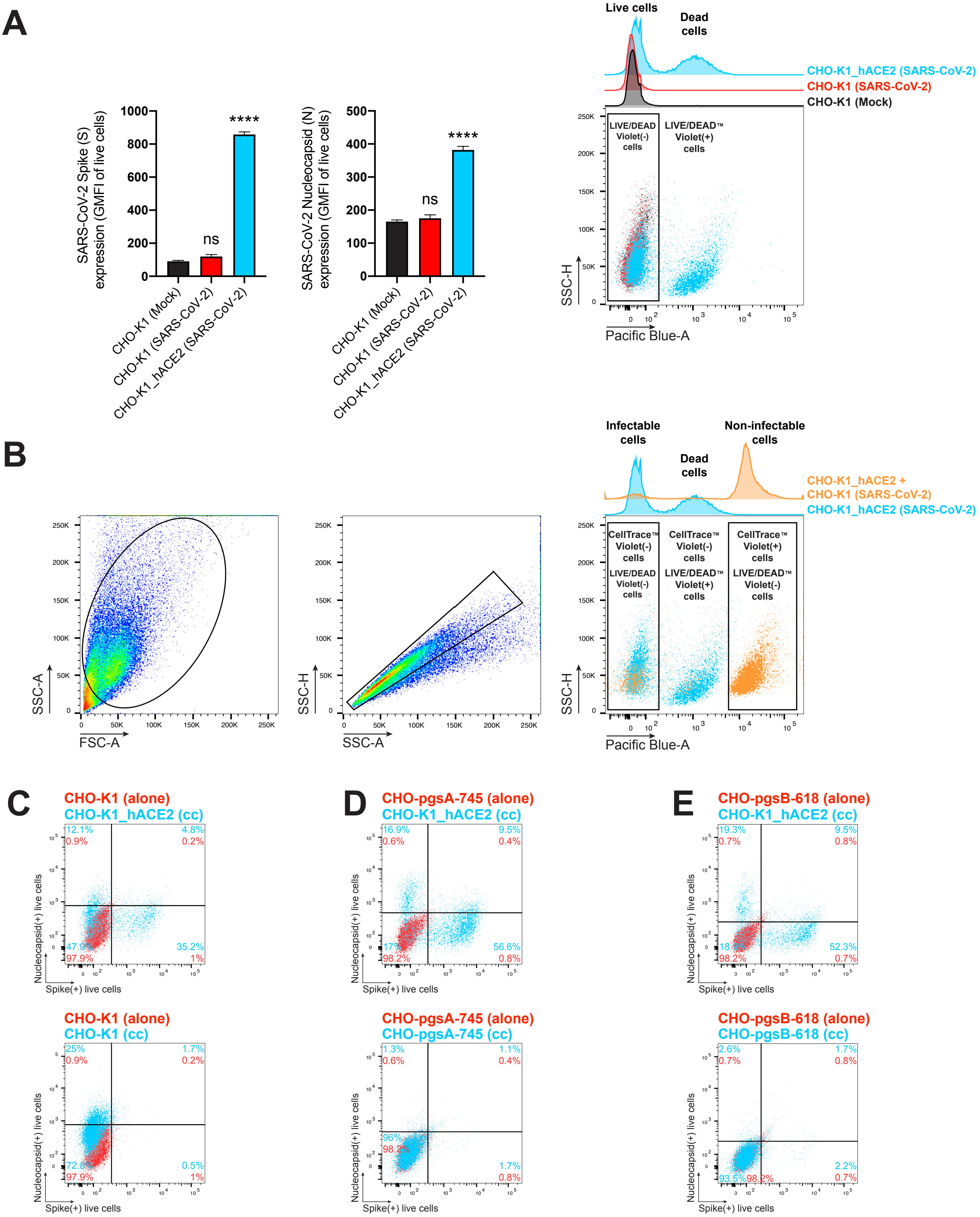

Fig. S8. Controls, gating strategies, and additional data for N transfer assays between SARS-CoV-2 infectable and non-infectable co-cultured cells. (**A**) Flow cytometry analyses of CHO-K1 and CHO-K1_hACE2 cells mock or SARS-CoV-2-infected (MOI = 1) and stained live at 24 hpi with Abs against SARS-CoV-2 S and N proteins and with LIVE/DEAD Violet. The GMFI of live cells expressing S and N proteins is plotted, showing mean +/- SEM (n = 3). One-way ANOVA and Dunnett s Multiple comparison test were used to compare all conditions against mock-infected cells: *ns* (nonsignificant) $p > 0.05$, **** $p < 0.0001$. Representative plots and histogram semi-overlays of LIVE/DEAD Violet staining are shown, indicating the gating for live cells. One representative experiment of at least two independent experiments performed in triplicate is shown. (**B**) Gating and staining strategy for live/dead, and infectable versus non-infectable cell exclusion for flow cytometry analyses shown in **Fig. 3**. CHO-K1, CHO-pgsA-745 and CHO-pgsB-618 cells were stained with CellTrace Violet prior co-culture with CHO-K1_hACE2 cells. Co-cultured cells were inoculated with SARS-CoV-2 (MOI = 1) and stained live at 24 hpi with Abs against SARS-CoV-2 S and N proteins and with LIVE/DEAD Violet, and analyzed by flow cytometry. Representative plots and histogram semi-overlays of LIVE/DEAD Violet and CellTrace Violet staining are shown, indicating the gating for live CellTrace Violet(-) cells (left), infected dead CellTrace Violet(-) cells (center), and uninfected live CellTrace Violet(+) cells (right). One representative experiment of at least two independent experiments performed in triplicate is shown. (**C-E**) Representative plots of flow cytometry analyses showing double staining of surface S and N proteins from the same histogram overlays shown in **Fig. 3**, indicating the percentage of the gated cell population for each quadrant of the double staining.

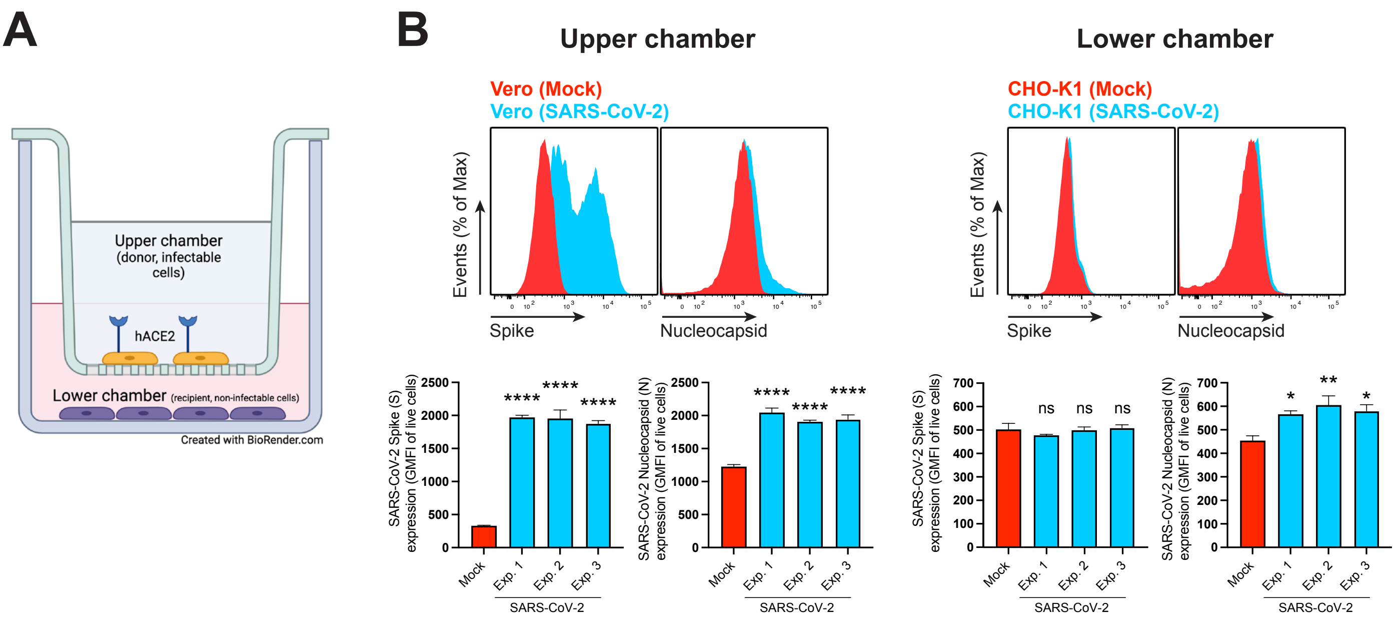

Fig. S9. In addition to cell-to-cell transference, SARS-CoV-2 N can be transferred by cell-free diffusion to neighboring uninfected cells. Flow cytometry analyses of N transference transwell assays between donor and recipient cells. (**A**) Schematic of the transwell assay. Non-infectable recipient CHO-K1 cells were plated in the lower chamber, while infectable donor cells in the upper chamber and infected here. (**B**) Histogram overlays of surface N and S proteins, as well as the GMFI of live cells expressing S and N proteins, from upper and lower chambers, is plotted showing mean +/- SEM (n = 3). Three independent experiments performed in triplicate are shown. One-way ANOVA and Dunnett s Multiple comparison test were used to compare each infection against mock-infected cells within each assay: *ns* (nonsignificant) $p > 0.05$, ** $p < 0.01$, *** $p < 0.001$, **** $p < 0.0001$.

A

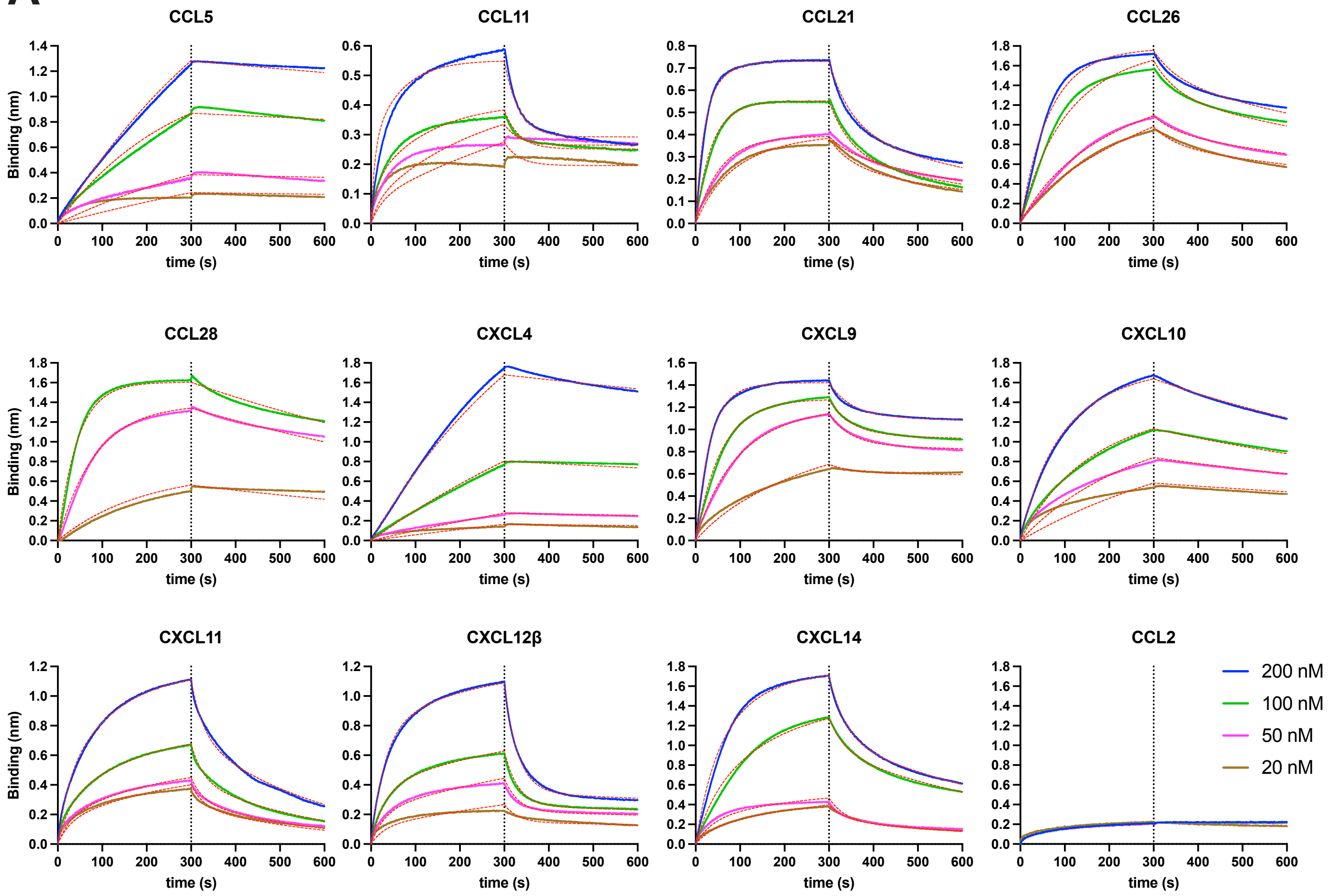

B

CHK	K_D (M)	k_{on} (1/M.s)	K_{off} (1/s)	R^2	Model
CCL5	$1\times10^{-8} \pm 1.5\times10^{-9}$	$1.9\times10^{4} \pm 6.5\times10^{2}$	$1.9\times10^{-4} \pm 2.7\times10^{-5}$	0.977	1:2
CCL11	$7.2\times10^{-8} \pm 7.3\times10^{-9}$	$1.1\times10^{5} \pm 4.4\times10^{3}$	$4.5\times10^{-3} \pm 1.7\times10^{-4}$	0.952	2:1
CCL21	$1.4\times10^{-8} \pm 1.9\times10^{-10}$	$2.7\times10^{5} \pm 1.9\times10^{3}$	$1.4\times10^{-3} \pm 1.9\times10^{-5}$	0.996	2:1
CCL26	$2.6\times10^{-8} \pm 6.5\times10^{-10}$	$6.3\times10^{4} \pm 5.7\times10^{2}$	$9.3\times10^{-4} \pm 1.1\times10^{-5}$	0.997	2:1
CCL28	$2.2\times10^{-8} \pm 9.3\times10^{-10}$	$1.2\times10^{5} \pm 8.5\times10^{2}$	$8.7\times10^{-4} \pm 1\times10^{-5}$	0.996	2:1
CXCL4	$1.7\times10^{-7} \pm 1.4\times10^{-8}$	$4.2\times10^{3} \pm 2.6\times10^{2}$	$5.3\times10^{-4} \pm 2.1\times10^{-5}$	0.997	1:2
CXCL9	$1.2\times10^{-8} \pm 1.6\times10^{-10}$	$1.2\times10^{5} \pm 5.4\times10^{2}$	$8.5\times10^{-4} \pm 7\times10^{-9}$	0.997	2:1
CXCL10	$1\times10^{-7} \pm 3.6\times10^{-9}$	$1.8\times10^{4} \pm 3.4\times10^{2}$	$1.6\times10^{-3} \pm 4.8\times10^{-5}$	0.994	1:2
CXCL11	$1.3\times10^{-7} \pm 5.3\times10^{-9}$	$4\times10^{4} \pm 8.1\times10^{2}$	$3.3\times10^{-3} \pm 3.6\times10^{-5}$	0.985	2:1
CXCL12β	$1.7\times10^{-7} \pm 1.2\times10^{-8}$	$2.2\times10^{4} \pm 9.6\times10^{2}$	$3.7\times10^{-3} \pm 4.9\times10^{-5}$	0.991	2:1
CXCL14	$4.5\times10^{-8} \pm 5.7\times10^{-10}$	$4.4\times10^{4} \pm 4.5\times10^{2}$	$1.9\times10^{-3} \pm 1.6\times10^{-5}$	0.996	2:1

Values reported represent the average of the global fit (± error) to the data from at least three independent BLI kinetic assays performed with different N protein preparations. Kinetics parameters were determined by using the heterogeneous ligand (2:1) model or the bivalent analyte (1:2) binding model.

Fig. S10. SARS-CoV-2 N protein specifically binds to 11 human chemokines with high affinity. (**A**) BLI sensorgrams of affinity kinetic assays between immobilized SARS-CoV-2 rN protein and positively bound human chemokines identified from BLI high-throughput-screening (HTS) binding assays. Sensorgrams show association and dissociation phases. The vertical dotted line indicates the end of the association step. Curves were analyzed with the ForteBio Data Analysis HT software, where red dashed lines correspond to a global fit of the data using the heterogeneous ligand (2:1) or bivalent analyte binding model (1:2). CCL2 is shown as example of negative interaction. All analyses were repeated with different protein preparations, and one representative assay out of at least three independent assays performed in duplicate is shown. (**B**) Table showing averaged values from the kinetic analyses of the N protein binding to each chemokine by BLI.

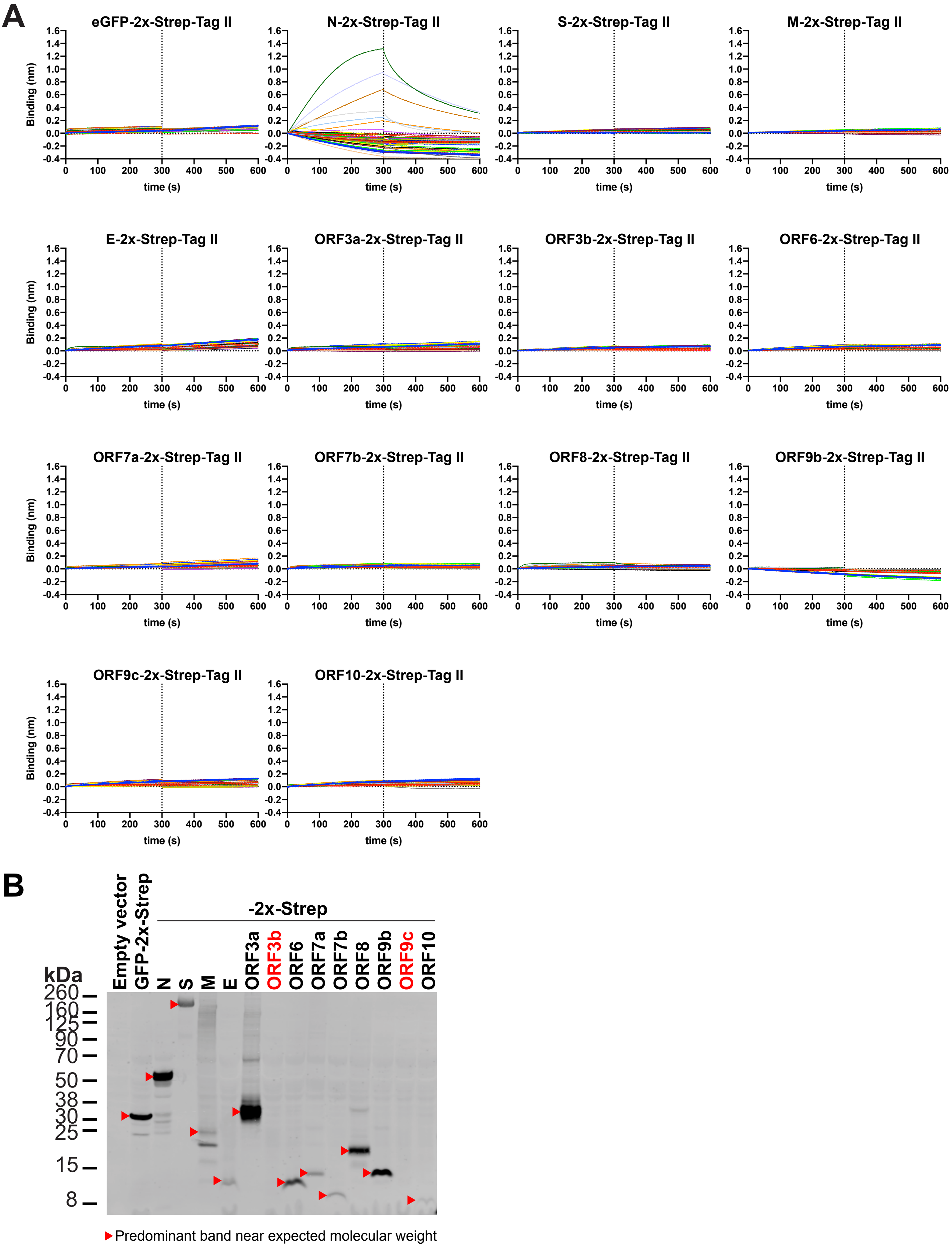

Fig. S11. Among SARS-CoV-2 structural proteins and accessory factors SARS-CoV-2 N protein uniquely binds chemokines. (**A**) BLI sensorgrams of HTS binding assays between immobilized eGFP, and SARS-CoV-2 structural proteins and accessory factors against 64 human cytokines at 100 nM (see detailed list in Material and Methods). N protein bound CCL5, CCL11, CCL21, CCL26, CCL28, CXCL4, CXCL9, CXCL10, CXCL11, CXCL12β and CXCL14 across different independent assays. Sensorgrams show association and dissociation phases. The dotted line indicates the end of the association step. The analyses were repeated with different protein preparations and one representative assay of two independent HTS is shown. (**B**) Immunoblot detection of 2xStrep tag verified expression of predicted protein sizes (red arrowheads), which were loaded into streptavidin-coated biosensors for BLI HTS binding assays. Despite the lack of detection of ORF3b and ORF9c (labeled in red) by immunoblot, the expression of these ORFs was detected after positive loading into streptavidin-coated biosensors by BLI. For gel source data, see **Fig. S14**.

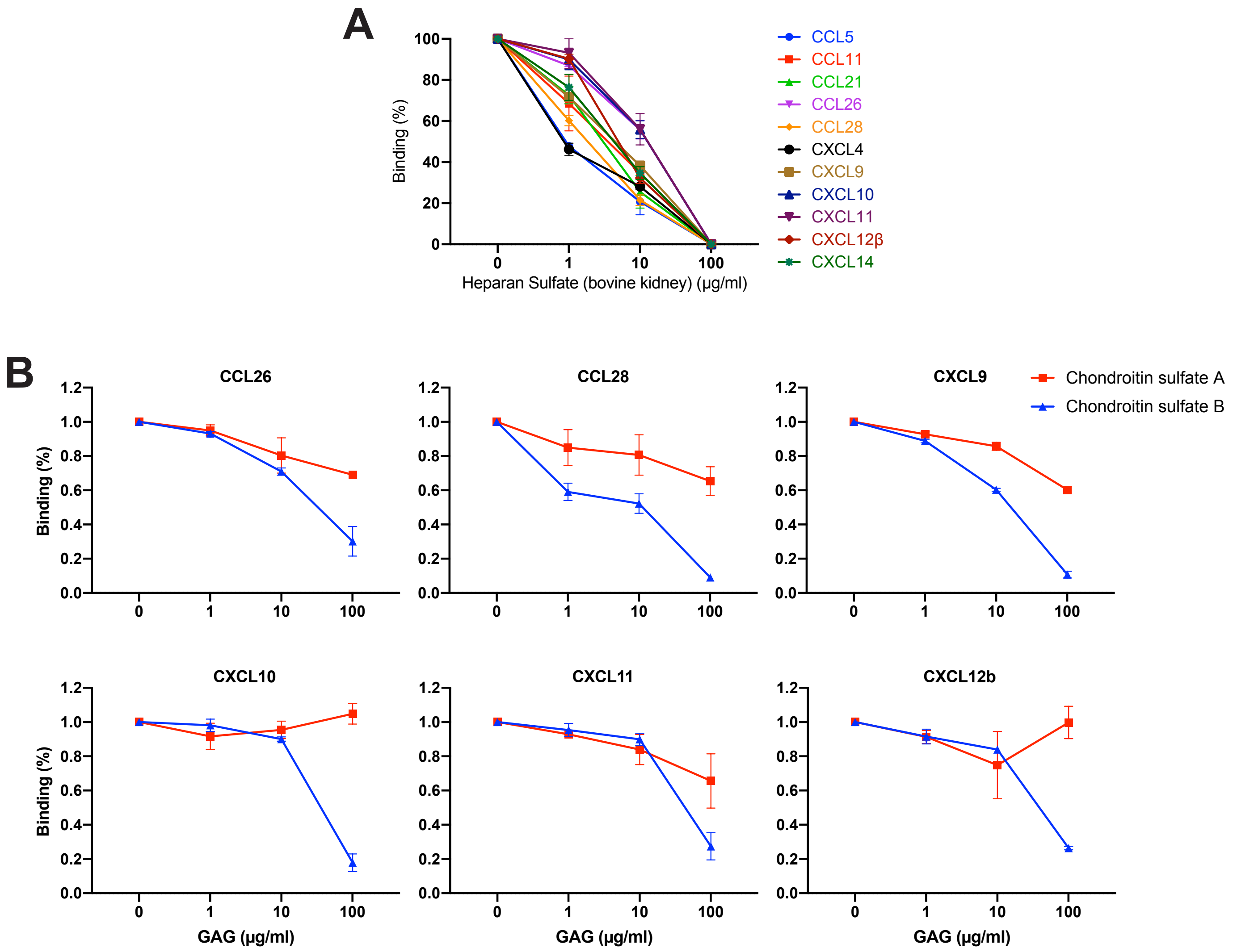

Fig. S12. SARS-CoV-2 N protein binds chemokines through the GAG-binding domain of chemokines. Sulfated GAG competition of chemokine binding to N protein. Chemokines at a concentration of 100 nM, alone or incubated with the indicated increasing concentrations of heparan sulfate from bovine kidney (**A**) or chondroitin sulfate A/B (**B**), were used for BLI binding analyses to N protein. The value of each chemokine binding without GAGs was considered 100%. Data represent the mean +/- SEM of 2-3 independent experiments. Heparan sulfate (from bovine kidney) interaction with N is considered negligible based on results from BLI assays shown in **Fig. 2F**.

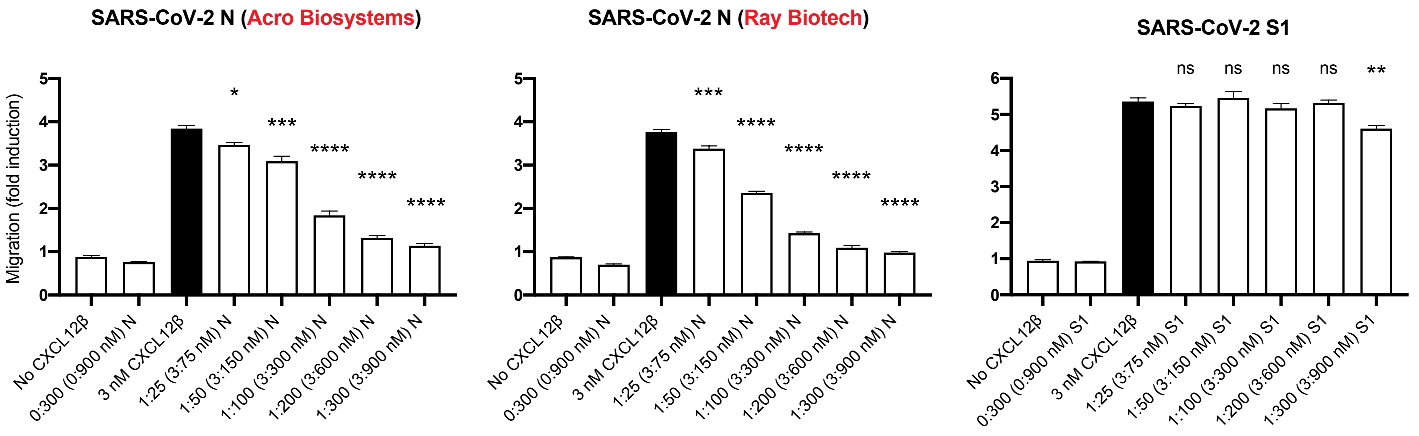

Fig. S13. Additional data supporting in vitro inhibition of chemokine-mediated leukocyte migration mediate by SARS-CoV-2 N but not S. Chemotaxis of MonoMac-1 cells induced by CXCL12β alone or in the presence of an increasing molar ratio of chemokine : viral protein. In all experiments, CXCL12β was incubated alone or in the presence of SARS-CoV-2 N or S1 (subunit 1 of the S protein) in the lower chamber of transwell migration devices. Migrated cells, added at the beginning of each experiment to the top chamber, were detected in the lower chamber at the end of the experiment. The induction of migration shows means +/- SEM (n = 3) from one representative assay performed in triplicate out of at least three independent assays. One-way ANOVA and Dunnett's Multiple comparison test were used to compare all conditions (except no chemokine and viral protein alone conditions) against migration induced by chemokine alone (dark bars): *ns* (nonsignificant) $p > 0.05$, * $p < 0.05$, ** $p < 0.01$, *** $p < 0.001$, **** $p < 0.0001$.

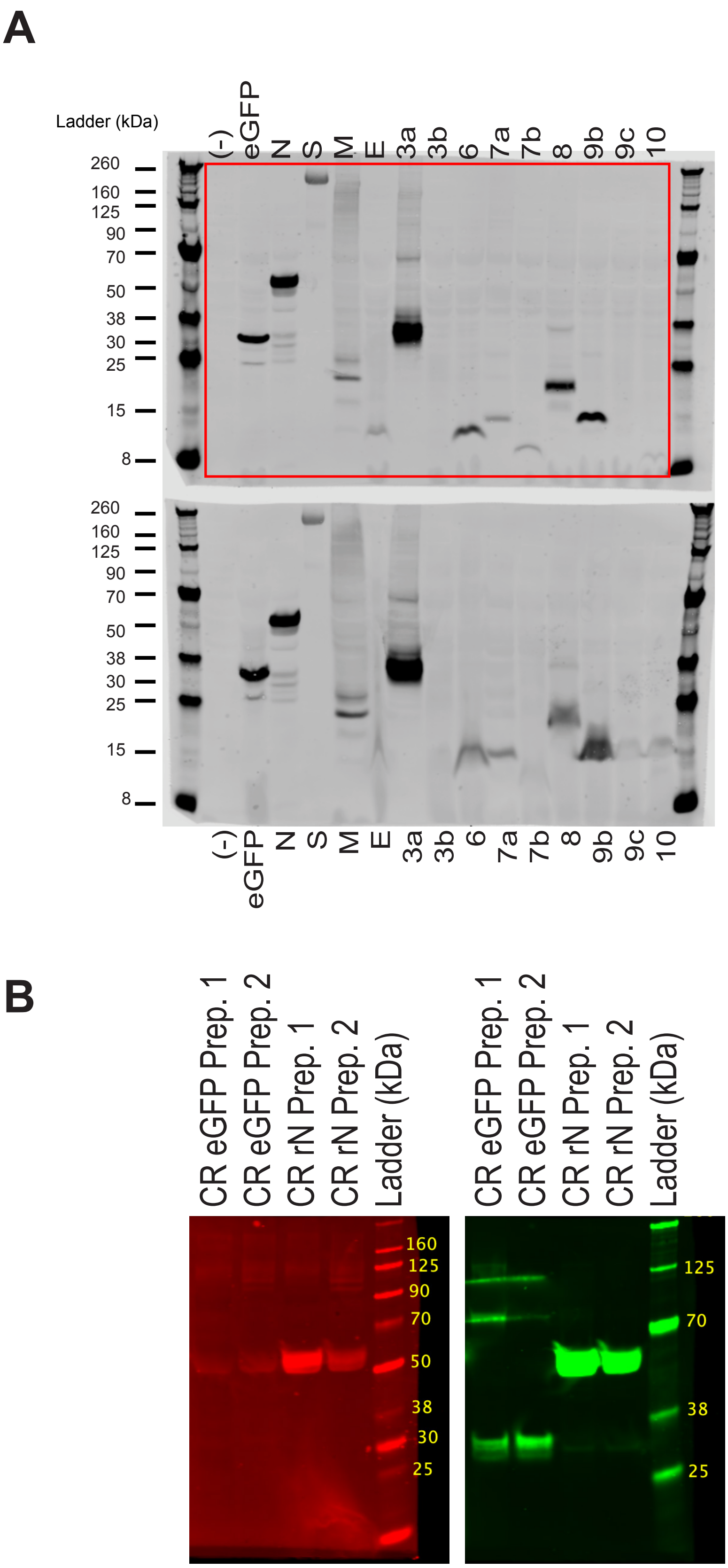

Fig. S14. Immunoblotting data. (**A**) Unprocessed immunoblot image with molecular weight ladder and red box outlining the image cropping used to generate **Fig. S11B**. Each blot corresponds to different protein preparations used for BLI assays. Both blots were stained with primary antibody mouse anti-2xStrep tag (Qiagen #34850, 1:1,000), and secondary goat anti-mouse IgG IRDye 800CW (LI-COR # 926-32210, 1:10,000). (**B**) Western blot image with molecular weight ladder from two different preparations of crude lysates (CR, 10 ul loaded) from transfected cells containing recombinant eGFP-2xStrep tag and N-2xStrep tag. The same blot was stained with IRDye 680RD Streptavidin (LI-COR # 926-68079), and human anti-SARS-CoV-2 N mAb (N18) followed by IRDye® 680RD Goat anti-Human IgG Secondary Ab (LI-COR # 926-68078).
