# Supplementary figures and images for: Cell surface SARS-CoV-2 nucleocapsid protein modulates innate and adaptive immunity
Source: Sci Adv. 2022 Aug 3;8(31):eabp9770. doi: 10.1126/sciadv.abp9770 (PMC9348789; doi:10.1126/sciadv.abp9770)

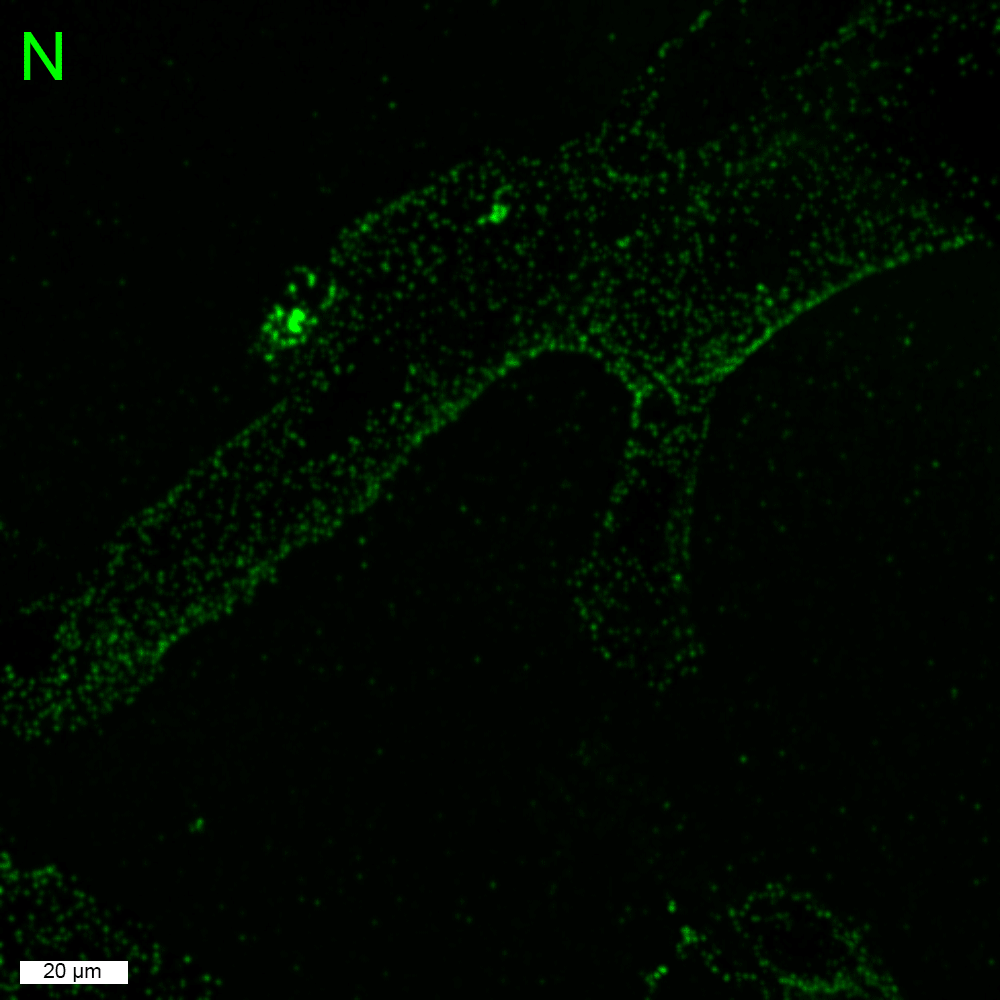

Supplement: Supplementary file 3 — Animations S1 to S14 [file sciadv.abp9770_animations_s1_to_s14.zip › sciadv.abp9770_animations_s1_to_s14/Animation S1. SARS-CoV-2 wt Vero cells.gif]

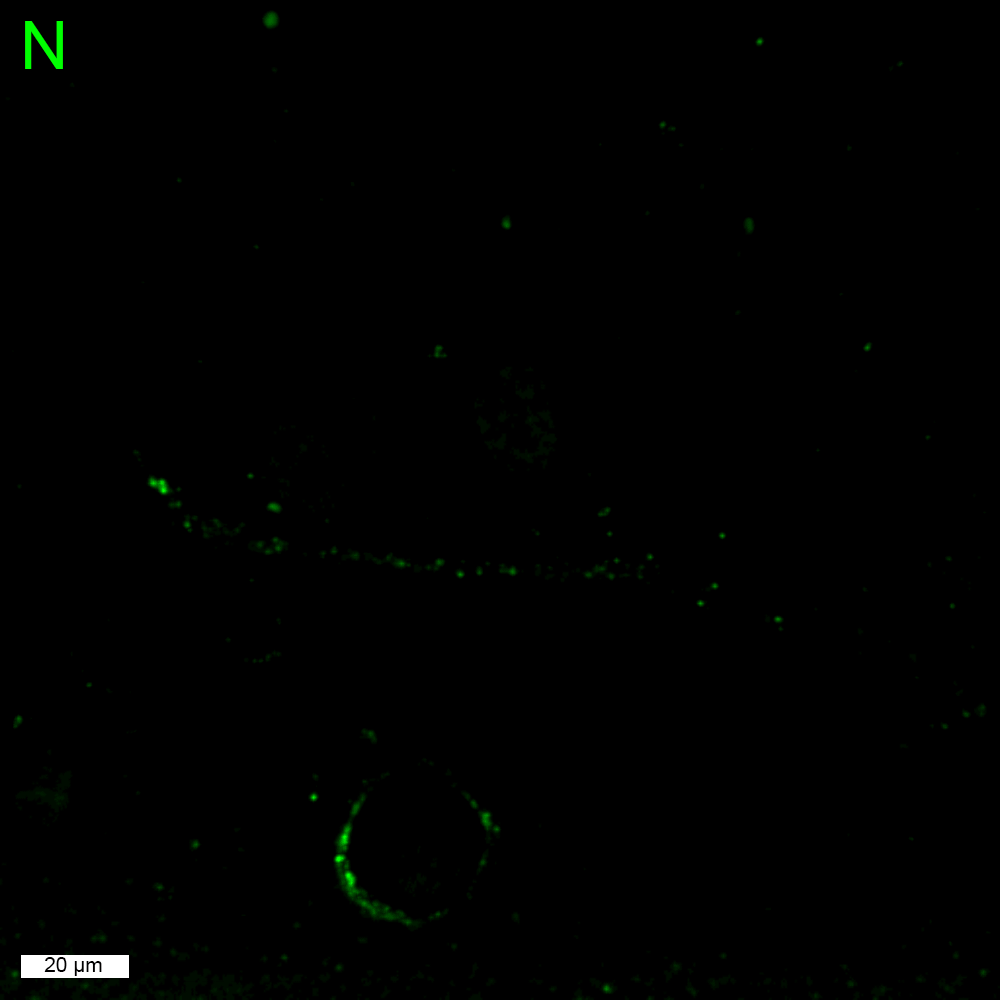

Supplement: Supplementary file 3 — Animations S1 to S14 [file sciadv.abp9770_animations_s1_to_s14.zip › sciadv.abp9770_animations_s1_to_s14/Animation S10. SARS-CoV-2 eGFP Caco-2 cells.gif]

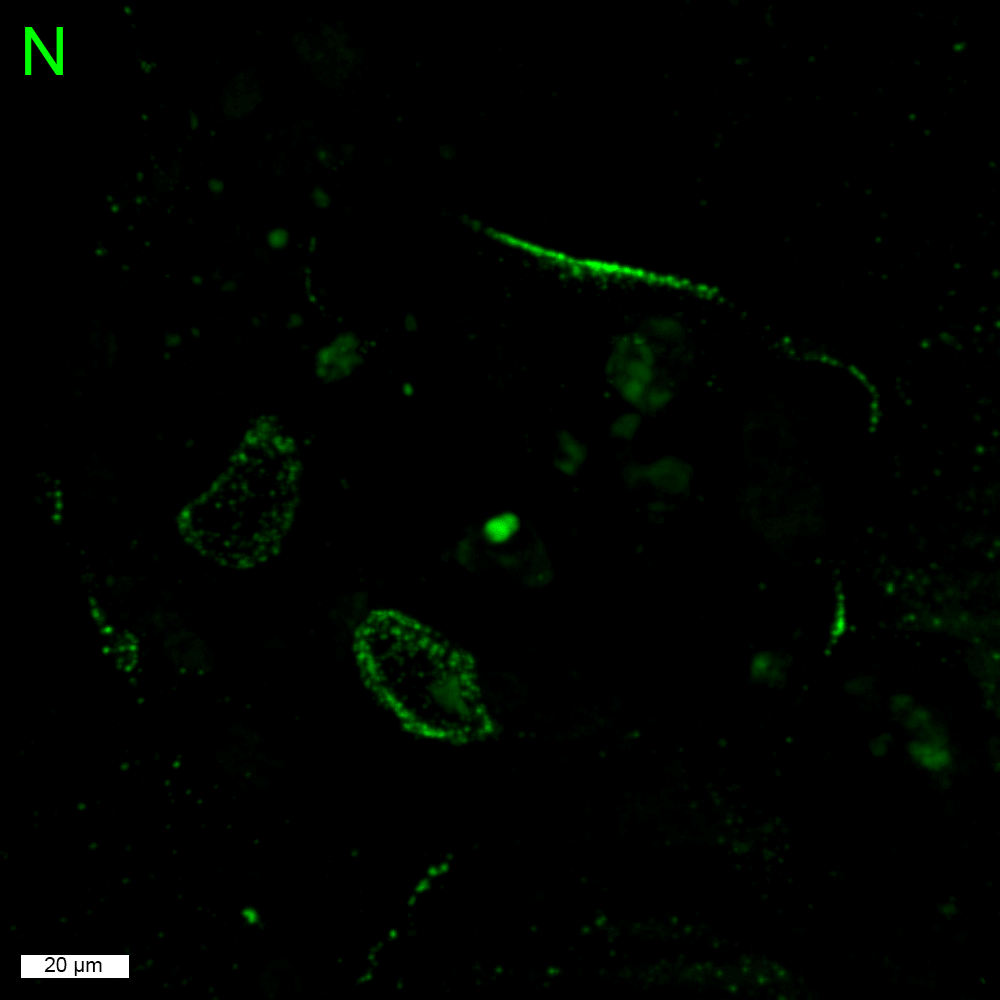

Supplement: Supplementary file 3 — Animations S1 to S14 [file sciadv.abp9770_animations_s1_to_s14.zip › sciadv.abp9770_animations_s1_to_s14/Animation S11. SARS-CoV-2 eGFP Calu-3 cells.gif]

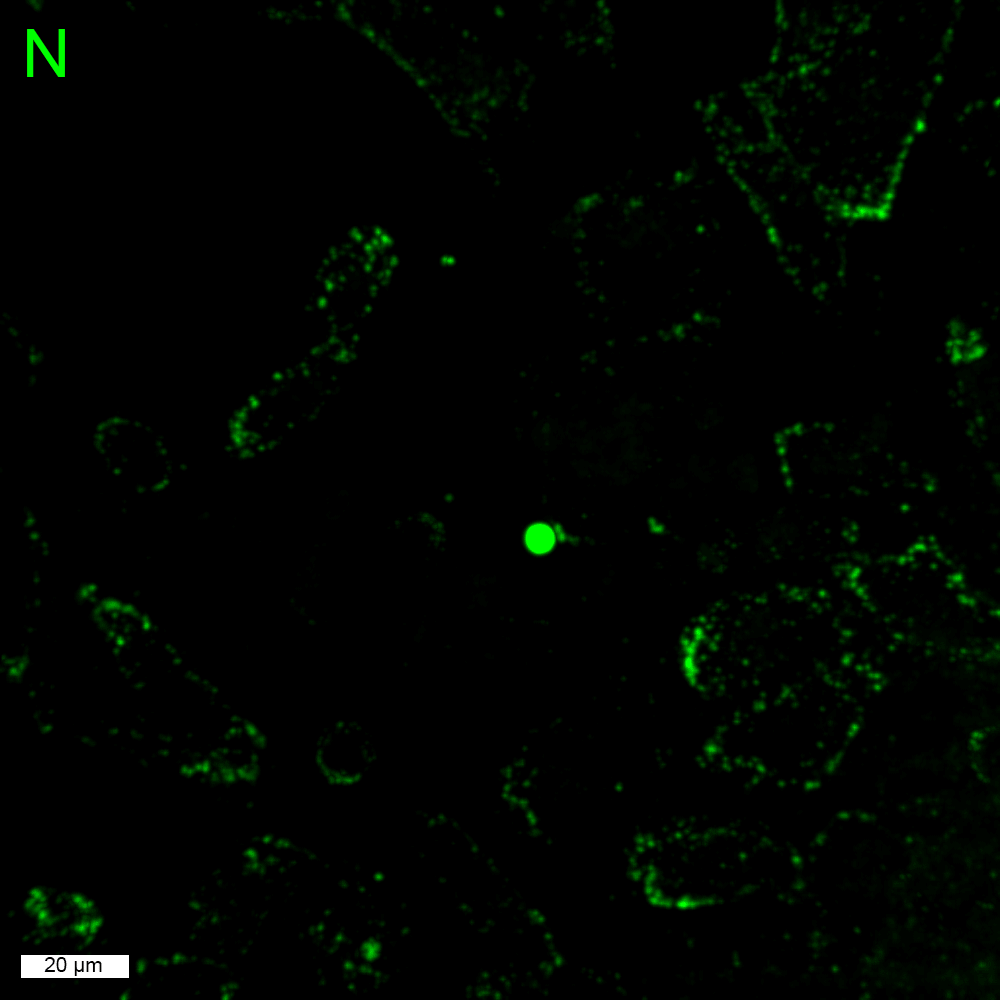

Supplement: Supplementary file 3 — Animations S1 to S14 [file sciadv.abp9770_animations_s1_to_s14.zip › sciadv.abp9770_animations_s1_to_s14/Animation S12. SARS-CoV-2 eGFP CHO-K1_hACE2 cells.gif]

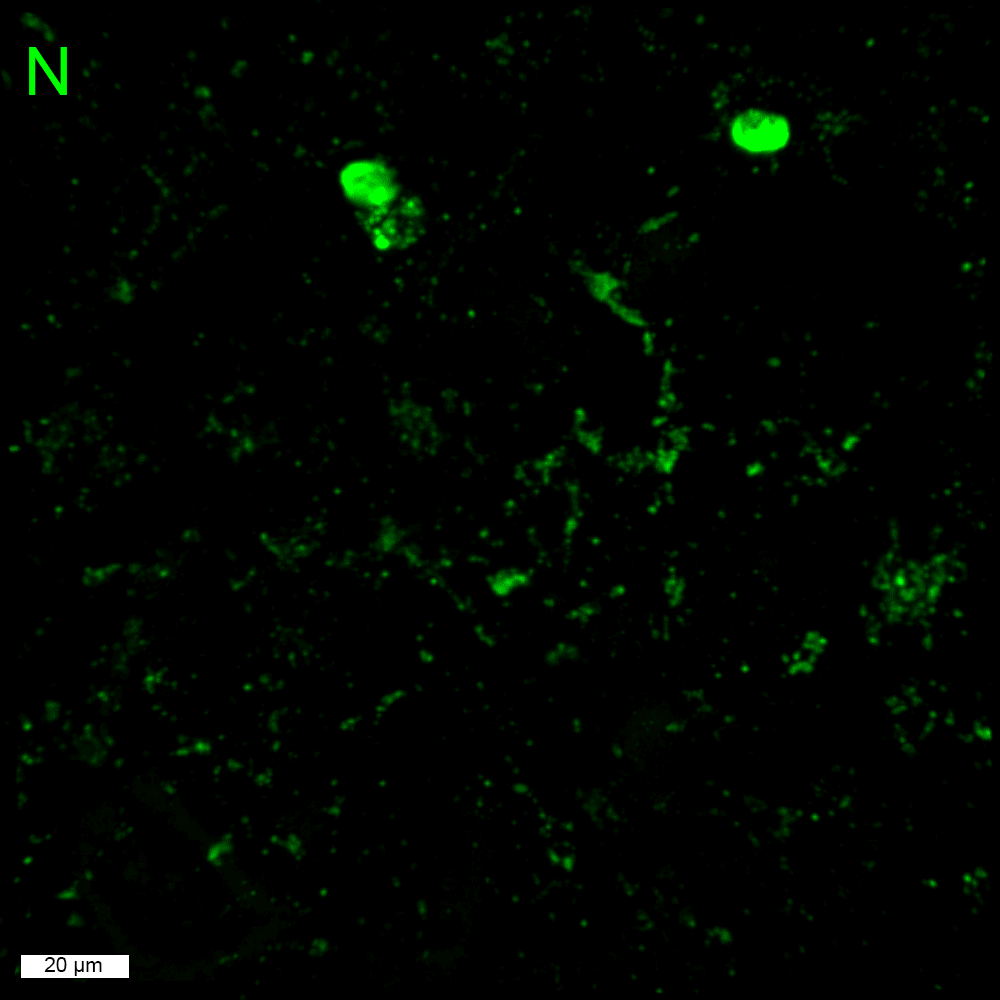

Supplement: Supplementary file 3 — Animations S1 to S14 [file sciadv.abp9770_animations_s1_to_s14.zip › sciadv.abp9770_animations_s1_to_s14/Animation S13. SARS-CoV-2 eGFP HEK293-FT_hACE2 cells.gif]

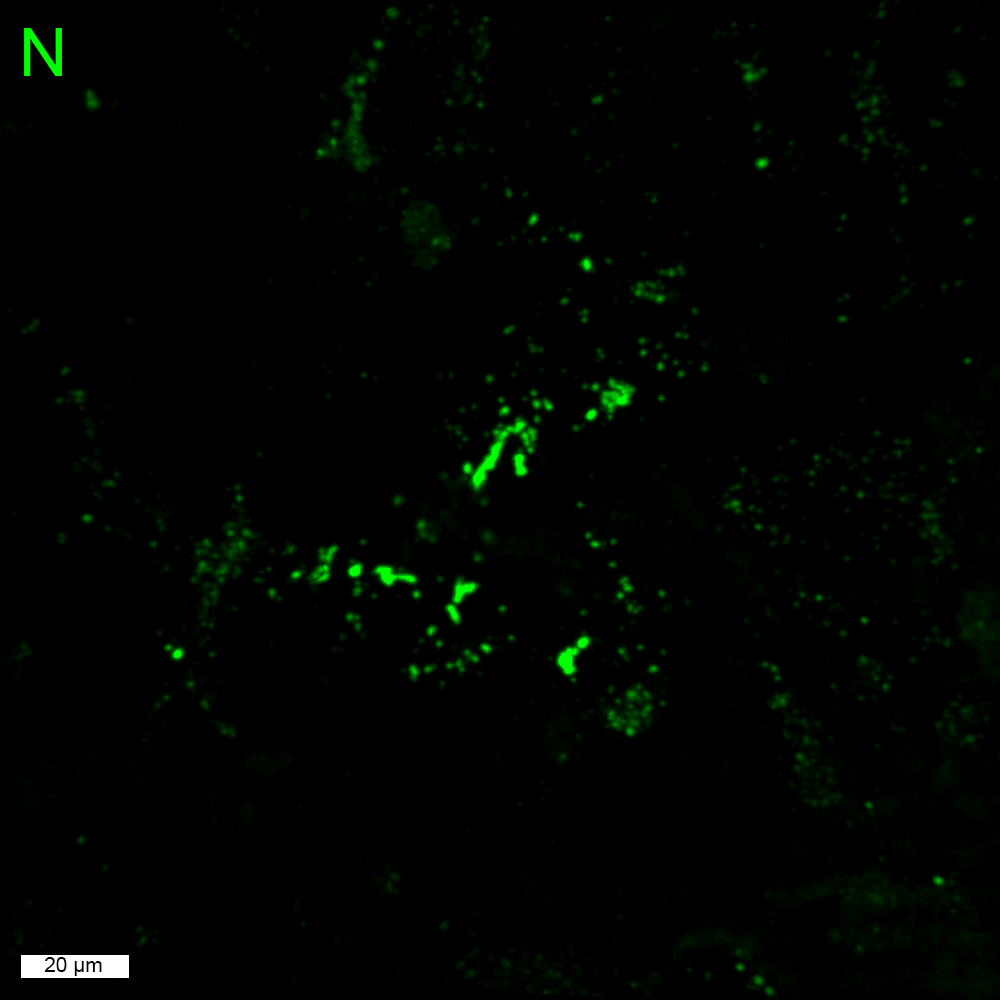

Supplement: Supplementary file 3 — Animations S1 to S14 [file sciadv.abp9770_animations_s1_to_s14.zip › sciadv.abp9770_animations_s1_to_s14/Animation S14. SARS-CoV-2 eGFP A549_hACE2 cells.gif]

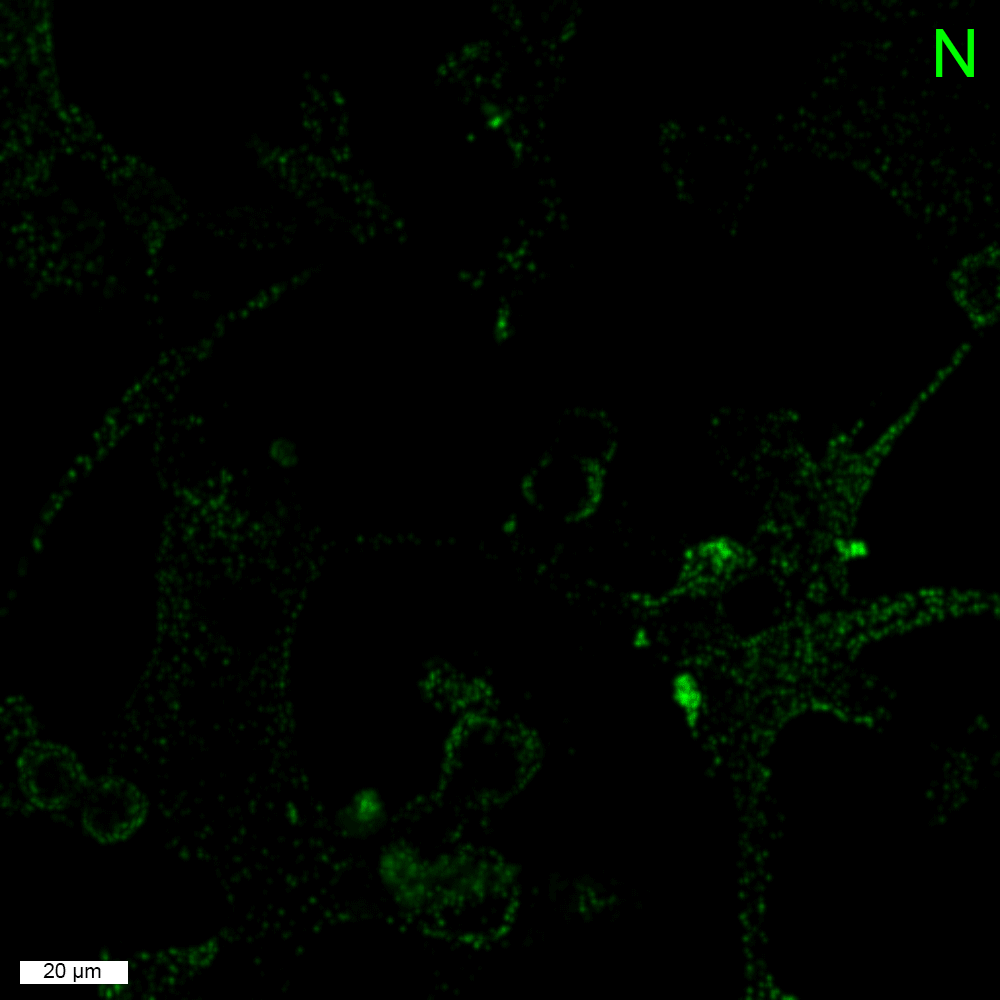

Supplement: Supplementary file 3 — Animations S1 to S14 [file sciadv.abp9770_animations_s1_to_s14.zip › sciadv.abp9770_animations_s1_to_s14/Animation S2. SARS-CoV-2 wt BHK-21_hACE2 cells.gif]

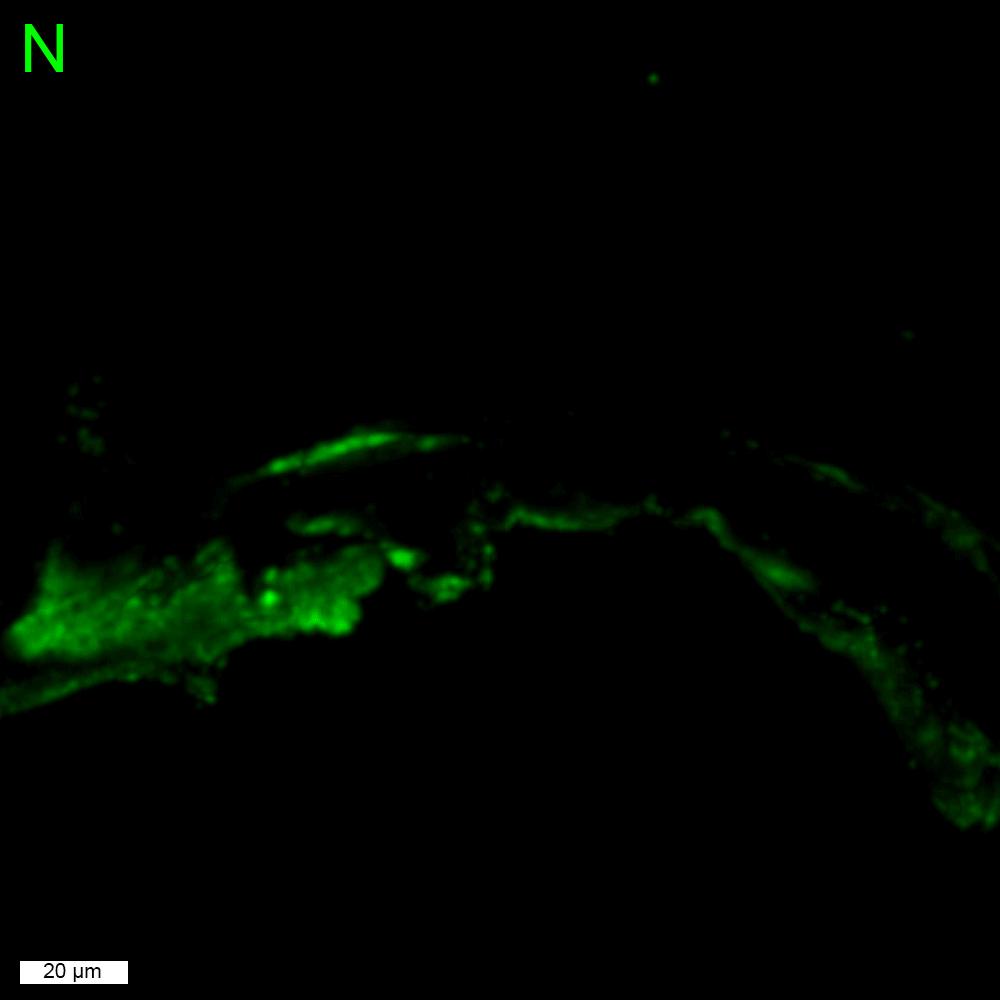

Supplement: Supplementary file 3 — Animations S1 to S14 [file sciadv.abp9770_animations_s1_to_s14.zip › sciadv.abp9770_animations_s1_to_s14/Animation S3. SARS-CoV-2 wt Caco-2 cells.gif]

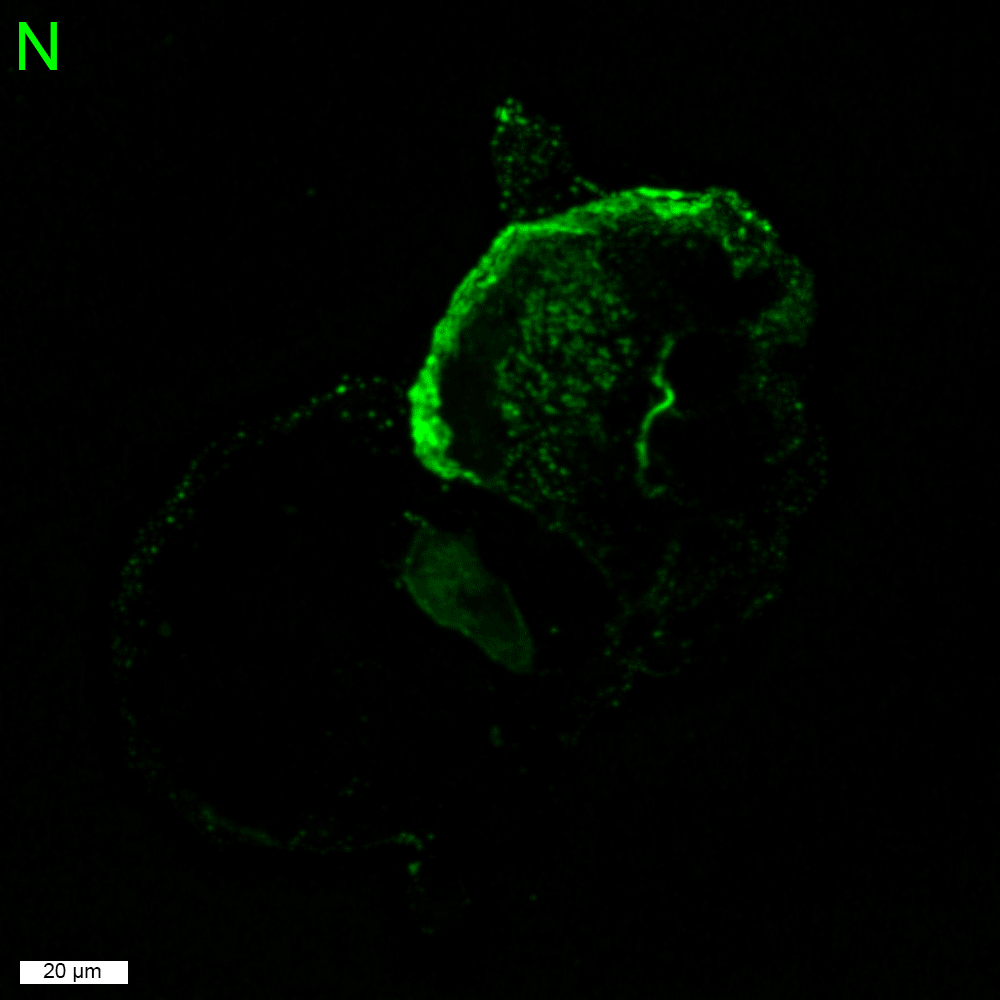

Supplement: Supplementary file 3 — Animations S1 to S14 [file sciadv.abp9770_animations_s1_to_s14.zip › sciadv.abp9770_animations_s1_to_s14/Animation S4. SARS-CoV-2 wt Calu-3 cells.gif]

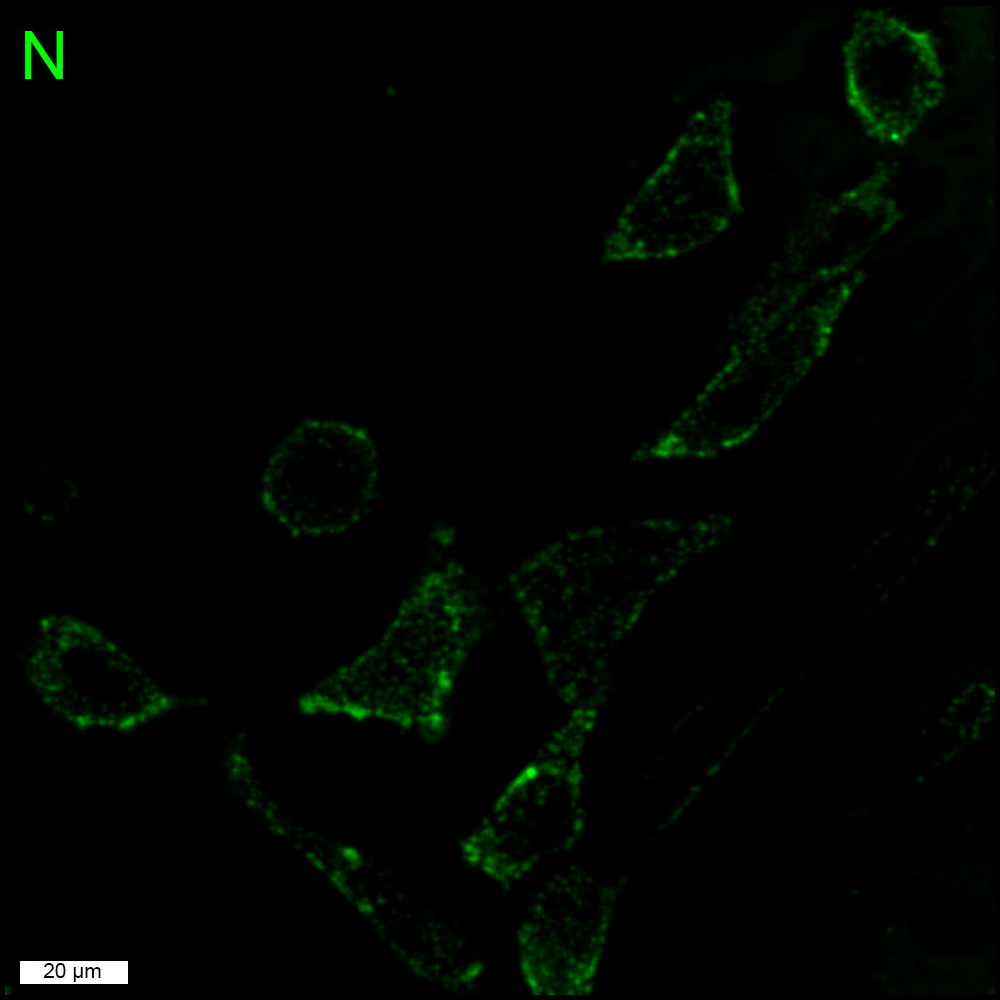

Supplement: Supplementary file 3 — Animations S1 to S14 [file sciadv.abp9770_animations_s1_to_s14.zip › sciadv.abp9770_animations_s1_to_s14/Animation S5. SARS-CoV-2 wt CHO-K1_hACE2 cells.gif]

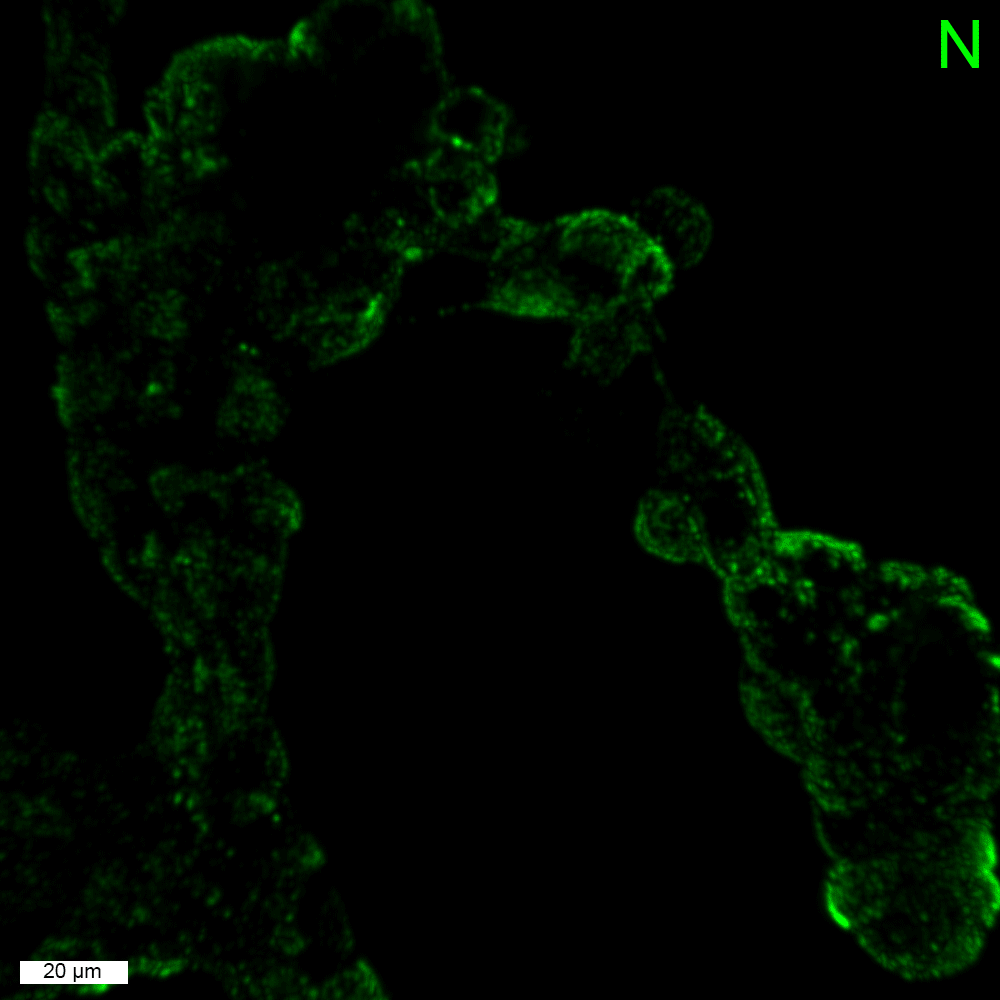

Supplement: Supplementary file 3 — Animations S1 to S14 [file sciadv.abp9770_animations_s1_to_s14.zip › sciadv.abp9770_animations_s1_to_s14/Animation S6. SARS-CoV-2 wt HEK293-FT_hACE2 cells.gif]

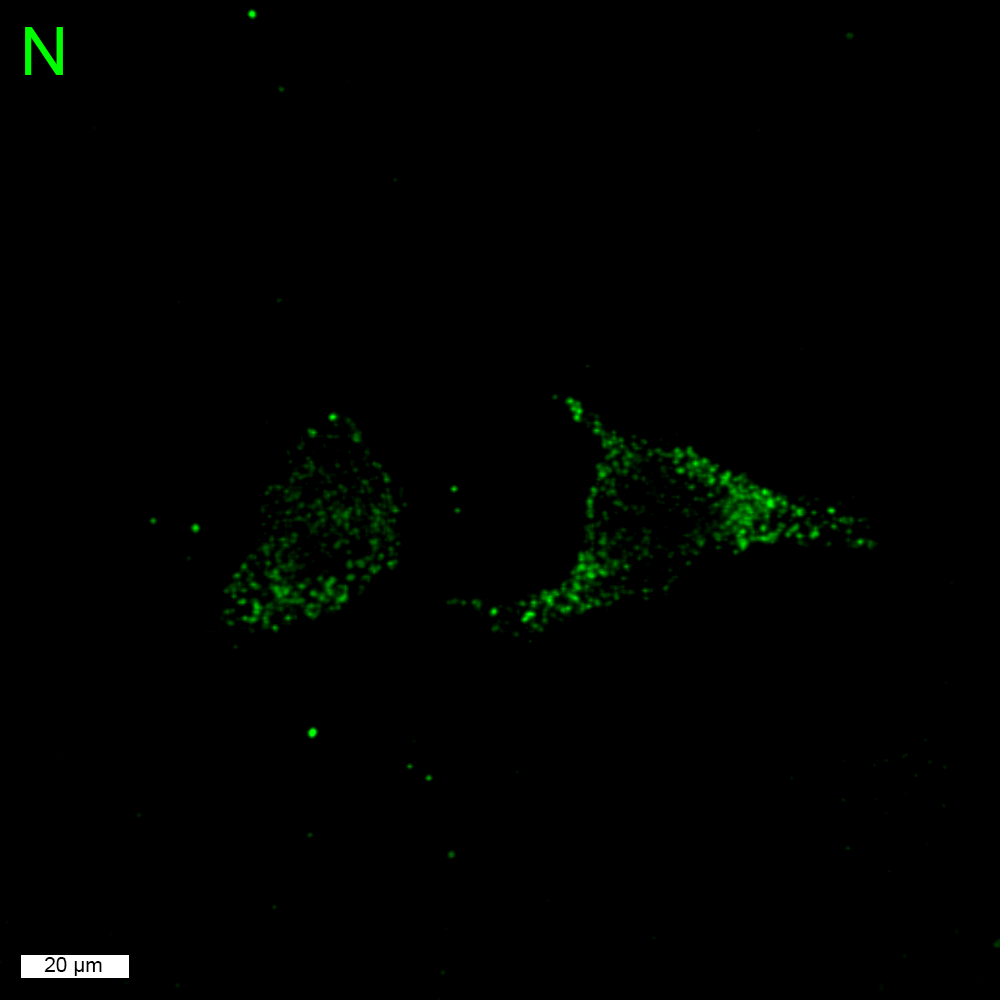

Supplement: Supplementary file 3 — Animations S1 to S14 [file sciadv.abp9770_animations_s1_to_s14.zip › sciadv.abp9770_animations_s1_to_s14/Animation S7. SARS-CoV-2 wt A549_hACE2 cells.gif]

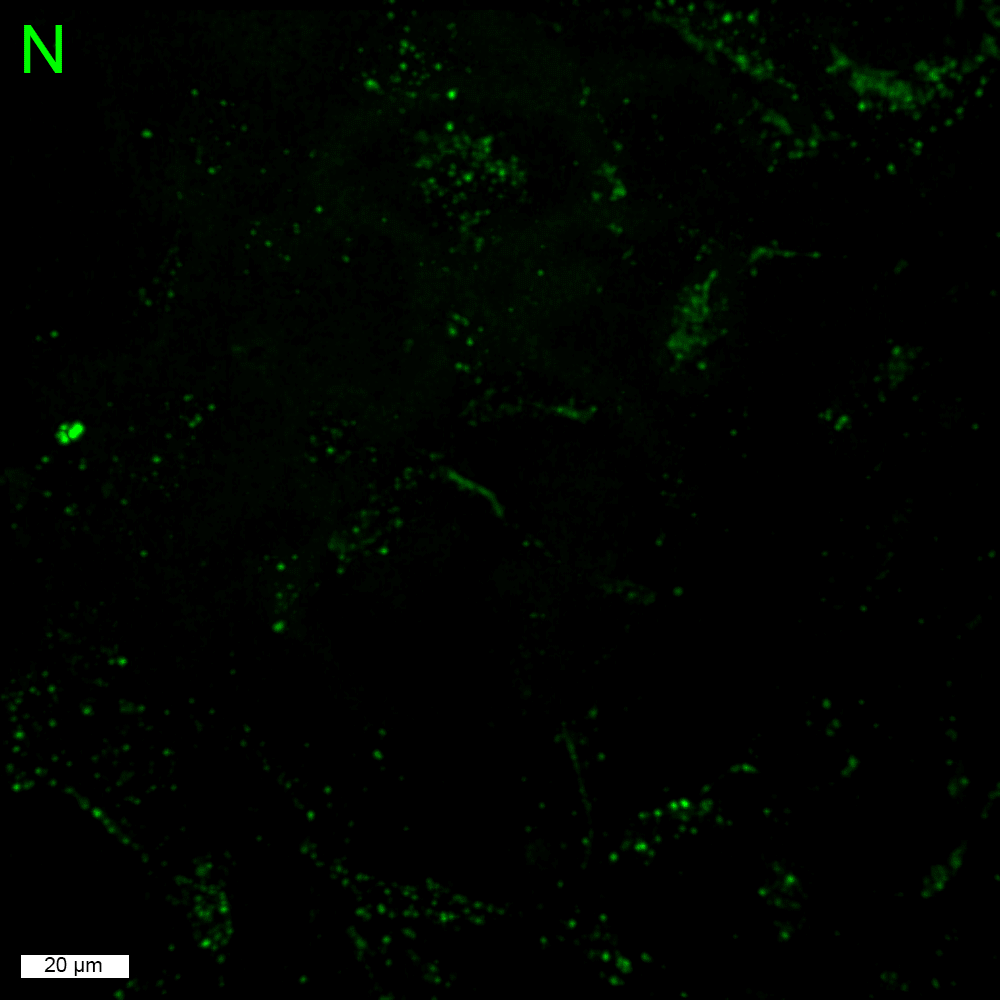

Supplement: Supplementary file 3 — Animations S1 to S14 [file sciadv.abp9770_animations_s1_to_s14.zip › sciadv.abp9770_animations_s1_to_s14/Animation S8. SARS-CoV-2 eGFP Vero cells.gif]

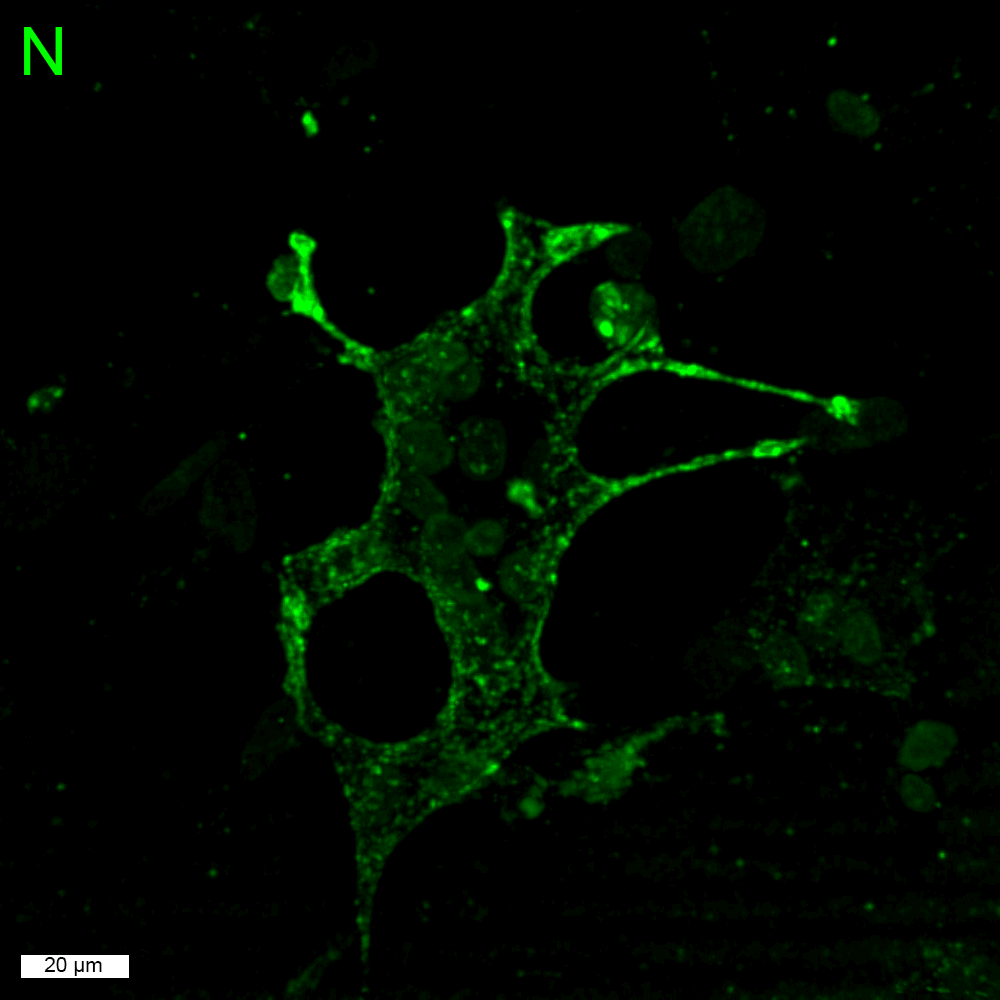

Supplement: Supplementary file 3 — Animations S1 to S14 [file sciadv.abp9770_animations_s1_to_s14.zip › sciadv.abp9770_animations_s1_to_s14/Animation S9. SARS-CoV-2 eGFP BHK-21_hACE2 cells.gif]
